# How antiretroviral data cleaning reshaped Mozambique’s progress to reaching HIV epidemic control

**DOI:** 10.1371/journal.pone.0354368

**Published:** 2026-07-29

**Authors:** Aleny Couto, Orrin Tiberi, Lindsay Templin, Morais da Cunha, Jose R. Mondlane, Jose Mizela, Hermínio Nhaguiombe, Ryan Keating, Ferreira Ferreira, Emilton Cumbana, Irénio Gaspar

**Affiliations:** 1 National Control Program for STIs and HIV/AIDS, National Public Health Directorate, Ministry of Health, Maputo, Mozambique; 2 Department of Interdisciplinary Social Science: Public Health, Utrecht UniversityNetherlands; 3 Division of Global HIV & Tuberculous, U.S. Centers for Disease Control and Prevention, Maputo, Mozambique; 4 U.S. Agency for International Development, Maputo, Mozambique; FHI 360, ZAMBIA

## Abstract

Quality data is fundamental for any public health program, especially for long-term care programs that rely on longitudinal data for decision making. In Mozambique, there had been a growing discrepancy between the number of people living with HIV (PLHIV) on antiretroviral treatment (ART) reported in the national system when compared to pharmacy and electronic medical records. A national ART data cleaning exercise was conducted in the first semester of 2024 to improve the accuracy and reliability of the nationally reported data. All health facilities with ART services (n = 1,757) compared their nationally reported data between the clinical data available at the health facility and the pharmacy medication pick-up data. The records that were successfully validated were marked as active, while the others were non-active. Upon completion of the review, the nationally reported data were revised. There was a 9.9% decrease (2,166,941–1,951,724) in the number of PLHIV considered currently on ART between December 2023 and June 2024. Among age groups, there was a 28.7% decrease among those less than 10 years of age, 26.4% among those between 10 and 19 years, and 8.0% for those 20 years or more. Cabo Delgado was the province with the largest percentage difference (21.1%, −28,799 PLHIV currently on treatment), while Zambezia had the largest absolute decline, of 41,445 (−8.9%). The notable decreases in PLHIV on ART resulted in an increase in Mozambique’s estimates for new infections, AIDS-related deaths, and vertical transmission. These updates allowed for a more realistic understanding of the HIV epidemic in Mozambique, including highlighting provinces with large data quality issues evidenced by substantial changes in PLHIV on ART numbers. This activity highlighted challenges that exist in paper-based reporting systems and provides evidence for a renewed focus on improving data quality for decision making.

## Introduction

The Joint United Nations Programme on HIV/AIDS (UNAIDS) has recognized data as one of the three critical cross-cutting areas to inform national HIV responses [1]. One of the most important indicators for monitoring the performance of HIV programs globally is the number of people living with HIV (PLHIV) currently receiving antiretroviral therapy (ART). This indicator is crucial for the UNAIDS HIV estimation model, Spectrum, which provides data on a country’s progress towards achieving epidemic control as defined by the 95-95-95 targets [2,3]. These targets were set to achieve HIV epidemic control, with 95% of PLHIV knowing their HIV status, 95% of those with known serostatus on ART, and 95% of those on ART with a suppressed viral load. On a yearly basis Mozambique develops and publishes Spectrum estimates that help guide the HIV response in the country. These estimates are developed using multiple inputs, including country programmatic data, national and international surveys, census data, and recent research [3]. Mozambique uses the estimates to inform strategic planning, resource allocation, and program monitoring [3–5].

The National Control Program for STIs and HIV/AIDS in Mozambique is supported by the President’s Emergency Plan for AIDS Relief (PEPFAR), which provides financial and technical assistance to the Mozambican Ministry of Health (MoH) for the HIV response. With this support, the country has been able to initiate over two million people living with HIV on ART. Reporting is done through different mechanisms for PEPFAR and MoH. The number of PLHIV on ART is reported to PEPFAR through an electronic medical record (EMR) from 658 health facilities with high ART volume, mostly focused in urban or peri-urban settings, while the MoH relies on manual counts of paper-based client charts reported to an aggregated electronic system [6].

The manual nature of reporting poses significant challenges to maintaining accuracy across program areas in Mozambique [7–9]. A 2024 national data quality assessment (DQA) among health facilities with ART services (n = 347) revealed that 18% of the 1,926 client charts sampled lacked accompanying pharmacy records to confirm ART pick-up [10]. Routine program validations, including DQAs and data triangulations consisting of an in-depth comparison of individual level records against other data systems, suggested an increasing misalignment within the reported number of PLHIV on ART. At the end of 2023, there was an 11% difference in the number of PLHIV currently on ART reported between PEPFAR and the MoH, as well as a 19% difference between the national aggregate pharmacy ART consumption data and the MoH reported results [11,12]. The result of these challenges have already been seen, with data on HIV positivity among pregnant women removed from the UNAIDS estimates process due to continued quality issues [13].

To address this misalignment, the MoH undertook a data cleaning exercise in all health facilities with ART services. In this manuscript, we report on the process and impact of Mozambique’s 2024 ART data cleaning exercise. By describing the justification, process and results of the chart review process and the impact of these findings on Mozambique’s estimates for tracking progress to HIV epidemic control, we hope that other countries will be encouraged to institute a similar initiative.

## Methods

### Process of ART data cleaning exercise

**Fig 1:**
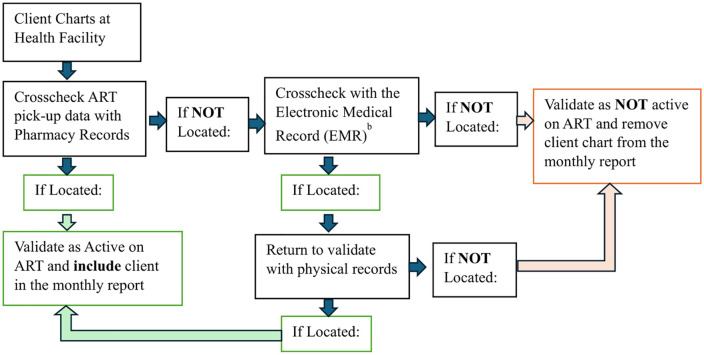
Data cleaning client chart validation workflow^a^. ^a^for the data cleaning, 100% of client charts are reviewed; for the supervision/validation, 10% of client charts are reviewed. ^b^only valid for health facilities with EMR, for those without EMR the client chart was considered NOT active if not located after the initial crosscheck.

The ART data cleaning exercise took place between December 2023 and June 2024 across 1,757 health facilities providing ART services in Mozambique. The HIV program in the MoH provided the orientation and tools for the health facility staff to review every client chart and crosscheck against pharmacy dispensation records to validate a client’s current engagement in ART. Following validation, the age of currently engaged clients was updated by data clerks based off date of birth and results were tallied into a revised total of PLHIV currently on ART. (Fig 1)

In cases where physical records were missing or unclear, facilities were authorized to consult the individual-level EMR and electronic pharmacy systems where available. Although these electronic systems were not officially used for validation purposes, they were instrumental in locating records, particularly in high-volume health facilities. Once verified, charts were then sorted according to updated status and ART registration number, effectively producing a clean set of active client records.

### Data analysis

To assess the impact of the data cleaning activity, we analyzed the absolute and percent change in the number of PLHIV on ART from results reported in December 2023 (pre-data cleaning) to results reported in June 2024 (post data-cleaning). To evaluate the reliability of the updated reported results, we calculated the change in the percent difference between PEPFAR-reported PLHIV on ART (reported from an EMR) and manually reported PLHIV on ART. PEPFAR defines PLHIV on ART as those currently receiving ART according to the national treatment protocol, not counting those who have not received ARVs ≤ 28 days from their last expected pickup, while the manual reported data uses a threshold of ≤59 days from last expected pickup visit [4]. Alignment of the reported results between the systems is considered reliable if the difference is less than 5%, a standard based on client-level analyses of the definitional differences [14]. Where possible, comparisons of interest were disaggregated by age (<10, 10–19, and 20 + years according to the priority age groups in the MoH reported data), sex (male and female), province, facility volume (1–500, 501–1000, 1000 + PLHIV on ART) and the presence of an EMR at the facility. Data access to the health facility level aggregated datasets at both MoH and PEPFAR occurred between the 23^rd^ and 27^th^ of June 2025. Of note, in the PEPFAR supplied data the military sites are aggregated due to privacy policies, while for the MoH reported data these sites are reported separately resulting in a different total of EMR sites between Table 1 and 2. Due to the aggregated nature of these datasets, no personal identifiers were accessed.

**Table 1:** Comparison of number of people with HIV currently on antiretroviral therapy (ART) before and after the 2024 data cleaning exercise, Mozambique (n = 1757 health facilities (HF)).

Indicator *(n = # of HF reporting)*		Pre-Data Cleaning (December 2023)	Post-Data Cleaning (June 2024)	Change (Pre-Post)	Percent Change^a^
**Total**		**2,166,941**	**1,951,724**	**−215,217**	**−9.9%**
**Reported Sex and Age Groups** **(*HF disag. not possible*)**	**<10 years**	81,817	58,341	−23,476	−28.7%
	**10-19 years**	134,228	98,776	−35,452	−26.4%
	**Female 10–19**	84,783	62,560	−22,223	−26.2%
	**Male 10–19**	49,445	36,216	−13,229	−26.8%
	**≥20 years**	1,950,896	1,794,607	−156,289	−8.0%
	**Female ≥20 years**	1,273,586	1,189,150	−84,436	−6.6%
	**Male ≥20 years**	677,310	605,457	−71,853	−10.6%
**Province**	**Niassa *(n = 206)***	66,397	55,829	−10,568	−15.9%
	**Cabo Delgado *(n = 109)***	136,670	107,891	−28,779	−21.1%
	**Nampula *(n = 231)***	290,745	249,488	−41,257	−14.2%
	**Zambezia *(n = 277)***	464,170	422,725	−41,445	−8.9%
	**Tete *(n = 153)***	127,268	117,077	−10,191	−8.0%
	**Manica *(n = 131)***	160,824	137,713	−23,111	−14.4%
	**Sofala *(n = 181)***	204,413	171,127	−33,286	−16.3%
	**Inhambane *(n = 151*)**	117,585	114,295	−3,290	−2.8%
	**Gaza *(n = 158)***	216,497	211,931	−4,566	−2.1%
	**Maputo Province *(n = 118)***	206,004	193,487	−12,517	−9.7%
	**Maputo City *(n = 42)***	176,368	170,161	−6,207	−3.5%
**Facility Volume**	**Low Volume (1–500) *(n = 944)***	178,495	177,254	−1,241	−0.7%
	**Medium Volume (501−1,000) *(n = 268)***	193,020	154,082	−38,938	−20.2%
	**High Volume (1,000+) *(n = 545)***	1,795,426	1,620,388	−175,038	−9.7%
**Presence of Electronic Medical Record at Facility**	**No *(n = 1,094)***	382,551	308,886	−73,665	−19.3%
	**Yes *(n = 658)***	1,784,390	1,642,838	−141,552	−7.9%

^**a**^ $Percent\;Change\;=\frac{Post\;Data\;Cleaning-Pre\;Data\;Cleaning}{Pre\;Data\;Cleaning}\;X\;\text{1}\text{0}\text{0}$

**Table 2:** Comparison between PEPFAR and Ministry of Health reports of the number of people with HIV (PLHIV) currently on antiretroviral treatment (ART) in Electronic Medical Records (EMR) health facilities (HF)s before and after the 2024 Data Cleaning, Mozambique (n = 639 healthcare facilities).

Indicator *(n = # of HF reporting)*		MOH PLHIV on ART v. PEPFAR PLHIV on ART Difference (Pre-Data Cleaning December 2023)				MOH PLHIV on ART v. PEPFAR PLHIV on ART Difference (Post-Data Cleaning June 2024)			
		PEPFAR PLHIV on ART	MOH PLHIV on ART	Abs. Difference	Percent Difference^a^	PEPFAR PLHIV on ART	MOH PLHIV on ART	Abs. Difference	Percent Difference^a^
**Total**		**1,676,431**	**1,788,852**	**112,421**	**6.5%**	**1,625,972**	**1,642,838**	**16,866**	**1.0%**
**Reported Age Bands** **(*HF disag. not possible*)**	**<10 years**	46,289	65,738	19,449	34.7%	41,917	47,936	6,019	13.4%
	**10-19 years**	67,333	106,474	39,141	45.0%	64,326	79,644	15,318	21.3%
	**Female 10–19**	43,042	68,486	25,444	45.6%	39,916	51,001	11,085	24.4%
	**Male 10–19**	24,291	37,988	13,697	44.0%	24,410	28,643	4,233	16.0%
	**≥20 years**	1,562,809	1,616,640	53,831	3.4%	1,519,729	1,515,258	4,471	0.3%
	**Female ≥20 years**	1,047,683	1,052,615	4,932	0.5%	1,023,083	1,001,486	21,597	2.1%
	**Male ≥20 years**	515,126	564,025	48,899	9.1%	496,646	513,772	17,126	3.4%
**Province**	**Niassa *(n = 20)***	33,699	37,538	3,839	10.8%	33,814	34,939	1,125	3.3%
	**Cabo Delgado *(n = 33)***	82,369	93,066	10,697	12.2%	75,496	80,268	4,772	6.1%
	**Nampula *(n = 64)***	198,446	221,016	22,570	10.8%	173,971	193,351	19,380	10.6%
	**Zambezia *(n = 149)***	375,401	405,960	30,559	7.8%	382,375	373,688	8,687	2.3%
	**Tete *(n = 34)***	95,300	97,453	2,153	2.2%	95,811	92,455	3,356	3.6%
	**Manica *(n = 52)***	133,926	140,145	6,219	4.5%	121,443	121,163	280	0.2%
	**Sofala *(n = 47)***	147,554	150,030	2,476	1.7%	128,072	125,407	2,665	2.1%
	**Inhambane *(n = 50)***	82,403	83,956	1,553	1.9%	84,740	82,692	2,048	2.4%
	**Gaza *(n = 101)***	178,337	185,719	7,382	4.1%	181,310	181,635	325	0.2%
	**Maputo Province *(n = 59)***	165,486	182,793	17,307	9.9%	164,920	172,465	7,545	4.5%
	**Maputo City*(n = 29)***	150,313	157,663	7,350	4.8%	151,950	151,925	25	0.0%
	**Military *(n = 1)***	33,197	33,513	316	0.9%	32,070	32,850	780	2.4%
**Facility Volume**	**Low Volume (1–500) *(n = 48)***	14,497	15,057	560	3.8%	14,680	15,017	337	2.3%
	**Medium Volume(501−1,000) *(n = 108)***	81,394	83,606	2,212	2.7%	85,334	82,445	2,889	3.4%
	**High Volume (>1,000) *(n = 483)***	1,580,540	1,690,189	109,649	6.7%	1,525,958	1,545,376	19,418	1.3%

**^a^** $Percent\;Difference=\frac{\left|\text{PEPFAR PLHIV on ART}-\text{ MOH PLHIV on ART}\right|}{\frac{\left|\text{PEPFAR PLHIV on ART}+\text{ MOH PLHIV on ART}\right|}{\text{2}}}\times \text{1}\text{0}\text{0}$

### HIV spectrum estimates

The internal processes of the Spectrum software package, mathematical modeling tools used to generate HIV estimates, have been extensively described in the literature [3]. Spectrum outputs include estimates for the number of PLHIV, the 95-95-95 cascade, new HIV infections, and HIV-related deaths, as well as future projections. One of the essential inputs for the model is the number of PLHIV on ART. As the data errors in the archive were assumed to have accumulated over time, historical provincial ART data were calculated between 2020 and the updated 2024 PLHIV on ART. The year 2020 aligned with the last national recount of the HIV, and the absolute difference between PEPFAR and MoH was only 0.10%, or 1,598 individuals [6]. The absolute difference between the number of PLHIV on ART in 2020 and 2024 was distributed between the intermittent years and weighted according to new ART initiations for 2021 (28% of the total increase), 2022 (28%), and 2023 (25%). After this update, these new historical treatment data were entered into the model to produce the 2024 Spectrum estimates. These adjusted 2024 estimates were then compared to the 2024 projections from the previous year’s estimates to determine the impact of the ART archive cleaning on the number of PLHIV, the 95-95-95 cascade, new infections, and deaths.

### Human ethics and consent to participate

The study was conducted in accordance with the Declaration of Helsinki in accordance to protocol 104/CNBS/2024 approved by the Mozambique National Bioethics Committee for Health. This activity was reviewed by the U.S. Centers for Disease Control and Prevention, deemed research not involving human subjects, and was conducted consistent with applicable federal law and CDC policy. The data used in this analysis are owned by the Ministry of Health Mozambique and come from routine aggregate data reports, without any personally identifiable information.

## Results

### Results in the MoH reported data

At the conclusion of the data cleaning exercise, there was a 9.9% (n = 215,217) reduction in the number of PLHIV on ART between December 2023 (2,166,941) and June 2024 (1,951,724) in the national reporting system. A decrease of 28.7% (23,476) was seen among children aged 0–10 years, 26.4% for adolescents 10–19 years (35,452), and 8.4% (156,289) among adults aged ≥20 years. (Table 1) When disaggregated by sex among adolescents, the percentage difference was closely aligned between female (26.2%) and males (26.8%). However, the difference was nearly double among adults aged ≥20 years by sex; females decreased 6.6% and males 10.6%.

### Comparison between MoH data and PEPFAR at health facilities with EMR

At health facilities with an EMR, the post-review results indicate a greater alignment between the two reporting systems for PLHIV currently on ART, dropping from a 6.5% difference in December 2023 to a difference of 1.0% in June 2024 (Table 2). Throughout the review, PEPFAR’s count of PLHIV on ART decreased from 1,676,529–1,625,972, or a 1.5% decline, while the MoH’s count of PLHIV on ART in sites with EMR fell from 1,788,852–1,642,838 representing a 4.3% decrease. The decreases in these data were mitigated by the identification and enrollment of out-of-care PLHIV during the same period through established programs of the HIV program. In the first six months of 2024 there were 136,256 newly initiated PLHIV on ART, and an additional 85,533 PLHIV who returned to treatment after a documented care interruption [12]. Without these additional entries into ART services, the absolute decrease in PLHIV on ART would have been substantially more.

Focusing specifically on adolescents aged 10–19 years, the data reveal a discrepancy between the Figs reported by PEPFAR and the MoH in December 2023, with a difference of 45.0% (67,334 for PEPFAR and 106,474 for MoH). During the subsequent exercise this difference decreased to 21.3% in June 2024 (absolute difference of 15,318). The number of adolescent PLHIV reported to PEPFAR decreased 2.3% from 67,334–64,326, while the MoH saw a more substantial decline of 14.4% (106,474–79,644) in these sites.

### Results in the national estimates projection

As a result of the data cleaning exercise, national ART coverage among all PLHIV dropped from 89% to 87%, with the largest drop among children 0–14 years (79% to 55%), in the 2024 estimates (Table 3). Overall new HIV infections increased by 22.7% (17,000), AIDS-related deaths rose by 12.8% (5,000), and vertical transmission rates increased from 8.5% to 12.3%. Children 0–14 years emerged with the largest relative changes in the estimates. The updated HIV estimates show a 28.6% (40,000) increase in the number of children living with HIV, a 70.0% (7,000) rise in new infections and a 49.3% (3,300) increase in AIDS-related deaths.

**Table 3:** Comparison of Spectrum results between the 2024 projections and the 2024 estimates for selected indicators.

Year: 2024	Projections^a^ (Upper-Lower Bounds)	Estimates^a^ (Upper-Lower Bounds)	Change (Pre-Post)	Percent Change
**HIV Prevalence (15–49 years):**	**11.08** [10.52 - 11.68]	**11.46** [10.83 - 12.12]	**0.38**	**3.4%**
**Vertical Transmission (%)**	**8.5** [7.8–9.7]	**12.3** [9.7 - 14.4]	**3.8**	**32.3%**
**People living with HIV on ART**	**2 220 000**	**2 050 000**	**170,000**	**7.7%**
**ART Coverage:** ^**b**^	**88.7%**	**81.8%**	**−6.9%**	**−7.8%**
*Children (0–14 years):*	78.8%	54.6%	−24.2%	−30.7%
*Women (15 + years):*	91.7%	85.1%	−6.6%	−7.2%
*Pregnant Women (15 + years)*^*c*^	*91.4%*	*81.7%*	*−9.7%*	*−10.6%*
*Men (15 + years):*	85.2%	81.7%	−3.5%	−4.1%
**People living with HIV:** ^**a**^	**2 500 000** [2 300 0001 – 2 700 000]	**2 500 000** [2 300 0001 – 2 700 000]	**0**	**0.0%**
*Children (0–14 years):*	**140 000** [120 000 - 160 000]	**180 000** [150 000 - 200 000]	**40,000**	**28.6%**
*Adolescents (10–19 years)* ^*c*^	***140 000*** *[100 000 - 170 000]*	***160 000*** *[110 000 - 200 000]*	**20,000**	**14.3%**
*Women (15 + years):*	**1 500 000** [1 400 000 – 1 600 000]	**1 500 000** [1 400 000 – 1 700 000]	**0**	**0.0%**
*Men (15 + years):*	**830 000** [770 000 - 910 000]	**800 000** [730 000 - 880 000]	**−30,000**	**−3.6%**
**New Infections:** ^**a**^	**75 000** [59 000 - 94 000]	**92 000** [73 000 - 114 000]	**17,000**	**22.7%**
*Children (0–14 years):*	**10 000** [9 0001234567891011 – 12 000]	**17 000** [11 000 - 21 000]	**7,000**	**70.0%**
*Adolescents (10–19 years)* ^*c*^	***14 000*** *[3 000 - 39 000]*	***17 000*** *[3 000 - 29 000]*	**3,000**	**21.4%**
*Women (15 + years):*	**42 000** [32 000 - 53 000]	**49 000** [38 000 - 63 000]	**7,000**	**16.7%**
*Men (15 + years):*	**23 000** [17 000–29 000]	**26 000** [20 000–34 000]	**3,000**	**13.0%**
**Deaths:** ^**a**^	**39 000** [34 000 - 46 000]	**44 000** [37 000 - 52 000]	**5,000**	**12.8%**
*Children (0–14 years):*	**6 700** [4 700 − 8 500]	**10 000** [6 700–13 000]	**3,300**	**49.3%**
*Adolescents (10–19 years)* ^*b*^*c*	***2 500*** *[1 700 − 3 400]*	***3 100*** *[2 300 − 4 100]*	**600**	**24.0%**
*Women (15 + years):*	**17 000** [15 000 - 21 000]	**19 000** [16 000 - 23 800]	**2,000**	**11.8%**
*Men (15 + years):*	**15 000** [12 000 - 18 000]	**15 000** [12 000–17 000]	**0**	**0.0%**

^a^The projections were elaborated in 2023 with pre-cleaning data, while the estimates were elaborated in 2024 and use the post-cleaning data

^b^Due to rounding, the disaggregation of the total may not always sum to the total

^c^Adolescents and Pregnant Women are shown as separate groups; however, these populations overlap with other age/sex categories

## Discussion

The ART data cleaning exercise, carried out between June 2023 and December 2024, demonstrated measurable improvements in the accuracy and consistency of Mozambique’s national ART data. Within a 12-month period, the difference between the PEPFAR EMR-based reported data and the MoH paper-based reported data decreased from 6.5% to 1.0%. Led by the MoH and implemented by provincial teams, the process revealed discrepancies across reporting systems, providing critical insights into data management and client monitoring challenges that can inform future health information strengthening efforts. While the 6.9% decrease in estimate ART coverage may appear as a setback, the long-term benefits of accurate, reliable data far outweigh these temporary challenges.

The chart review revealed considerable discrepancies between previously reported Figs and revised data, exposing long-standing challenges in client monitoring, data management, and reporting systems. Paper-based records continue to undermine the integrity of data across the entire lifecycle. Data fragmentation and duplication are also pressing issues, often arising from unlinked health facility records or parallel reporting systems. These challenges may be behind the uneven geographic ART reductions seen in Table 1 and 2, which warrant further qualitative investigation to understand differences between provinces and learn best practices not captured in this exercise. Some potential factors identified anecdotally during the activity are provincial and/or district leadership, different clinical partner support structure, and even frequency of humanitarian disasters. Although initiatives to improve data systems are underway, progress is uneven and implementing a systemwide review may be beneficial for a unified national approach.

These adjustments have had a ripple effect on national Spectrum HIV estimates. The increase in new infections aligns with the lower ART coverage in the updated estimates, showing the impact of a higher number of undiagnosed and untreated individuals than previously estimated [15]. Increases in AIDS-related deaths may reflect a more accurate picture of mortality among PLHIV, including the higher risk of mortality among PLHIV with advanced HIV disease [16,17]. While increases in rates of vertical transmission point to continued challenges, especially in the coverage of prevention of mother-to-child transmission efforts among all HIV-positive pregnant women [18]. Among all demographic groups, the increases in estimates for adolescents living with HIV underscore the urgency of strengthening effective adolescent-focused interventions, including efforts to improve low ART coverage such as improving early diagnosis and ensuring long-term care retention for this priority population [19].

This exercise came at a time when the country committed to the achievement of the 95-95-95 targets by the end of 2025. Prior to the data cleaning in 2023, the national HIV care cascade was reported at 89-97-90 [7]. However, at the end of 2024 the cascade now reflects a more realistic but lower coverage rate of 87-94-91, directly influenced by the reduction in reported ART clients [12]. The adolescent-specific cascade presents an even graver situation, standing currently at 70-96-77. This highlights substantial gaps in adolescent HIV testing and retention, necessitating focused corrective measures. The updated cascades allow the country to have a more realistic understanding of the state of the HIV response and to set priorities for the coming years to ensure progress toward epidemic control and eventually ending HIV as a public health threat by 2030.

### Reflections

Even after the review, many of the challenges identified in the cleaning exercise persist. Recognizing the value of the data cleaning initiative, the MoH has recommended that this activity be institutionalized as a routine annual process, with structured oversight from district and provincial health authorities [20]. Institutionalizing targeted routine data cleaning exercises can yield several benefits, including up-to-date and accurate client records and more realistic national targets aligned with service delivery and population needs. These improvements are critical to a successfully coordinated national response and ultimately can improve the country’s ability to eliminate HIV as a public health threat. Routine data quality interventions, along with broader investments in health information systems that may allow integrated reporting between PEPFAR and the MoH, are core elements of national efforts to achieve HIV epidemic control. Achieving these outcomes requires investment in data quality, including human resource capacity, digital health infrastructure, and stronger monitoring and evaluation frameworks with clear indicators, regular supervision, and accountability mechanisms.

### Limitations

There are some important limitations to consider from this exercise. Limited resources prevented supervision visits to all facilities and data cleaning validations focused on larger, more centrally located health facilities. In contrast, rural and smaller health facilities without donor support, which make up most of health facilities with ART services, did not have regular supervision visits for validation despite recording the largest differences in the analysis. The results presented for these health facilities may be larger or smaller than reported depending on implementation fidelity, especially in provinces such as Inhambane and Gaza where the EMR was used for reporting cleaning data. In addition, data reporting in the national system can vary depending on many factors such as reporting error, climatic events and social unrest. Some discrepancies in reported PLHIV on ART from both before and after the exercise may have been impacted by these factors.

## Conclusions

The 2024 ART data cleaning exercise provided a more accurate foundation for monitoring Mozambique’s HIV response and highlights the importance of data quality, triangulation, and accuracy. Despite the discouraging impact on the HIV estimates, the Government of Mozambique recognized that more accurate data were essential for effective planning, prioritization, and accountability. The cleaning exercise created a more reliable baseline for program performance and target-setting, while exposing systemic weaknesses in the health information system previously obscured by incorrect data. In this sense, the chart review exercise played a pivotal role not only in revealing the true scope of the HIV epidemic but also providing the rationale towards increased focus on improving quality for data-driven decision-making.

## Supporting information

S1 File Data files for table 1 and 2 produced in this manuscript. (XLSX)
