# Supplementary material for: How antiretroviral data cleaning reshaped Mozambique’s progress to reaching HIV epidemic control
Source: PLoS One. 2026 Jul 29;21(7):e0354368. doi: 10.1371/journal.pone.0354368 (PMC13419232; doi:10.1371/journal.pone.0354368)
Supplement: Attachment — Submitted filename: PONE-D-26-12452.pdf [file pone.0354368.s002.pdf]

PLOS One

How antiretroviral data cleaning reshaped Mozambique’s progress to reaching HIV epidemic control

--Manuscript Draft--

Manuscript Number:	PONE-D-26-12452
Article Type:	Research Article
Full Title:	How antiretroviral data cleaning reshaped Mozambique’s progress to reaching HIV epidemic control
Short Title:	Impact of antiretroviral data cleaning in Mozambique
Corresponding Author:	Orrin Tiberi<br>Ministry of Health<br>Maputo, MOZAMBIQUE
Keywords:	HIV; Mozambique; HIV Estimates; Data Accuracy; Decision Making
Abstract:	Background: Quality data is fundamental for any public health program, especially for long-term care programs that rely on longitudinal data for decision making. In Mozambique, there had been a growing discrepancy between the number of people living with HIV (PLHIV) reported in the national system when compared to pharmacy and electronic medical records. A national ART data cleaning exercise was conducted in the first semester of 2024 to improve the accuracy and reliability of the nationally reported data.<br>Methods: All health facilities with ART services (n=1,754) triagulated their nationally reported data between the clinical data available at the health facility and the pharmacy medication pick-up data. The records that were successfully triangulated were marked as active, while the others were non-active. Upon completion of the review, the nationally reported data were revised.<br>Results: There was a 10.3% decrease (2,166,941 to 1,944,204) in the number of PLHIV considered currently on ART between December 2023 and June 2024. Among age groups, there was a 28.9% decrease among those less than 10 years of age, 26.6% among those between 10 and 19 years, and 8.4% for those 20 years or more. Cabo Delgado was the province with the largest percentage decline (21.1%, -28,799 PLHIV currently on treatment), while Zambezia had the largest absolute decline, of 41,445 (-8.9%).<br>Discussion: The decreases in ART coverage resulted in an increase in Mozambique's estimates for new infections, AIDS-related deaths, and vertical transmission. These updated allowed for a more realistic understanding of the HIV epidemic in Mozambique, including highlighting provinces with large data quality issues evidenced by substancial changes in PLHIV on ART numbers. This activity highlighted challenges that exist in paper-based reporting systems and provides evidence for a renewed focus on improving data quality for decision making.
Order of Authors:	Aleny Couto, MD
	Orrin Tiberi
	Lindsay Templin
	Morais da Cunha
	Jose R Mondlane
	Jose Mizela
	Herminio Nhaguiombe
	Ryan Keating
	Ferreira Ferreira
	Emilton Cumbana
	Irénio Gaspar, MD

Powered by Editorial Manager® and ProduXion Manager® from Aries Systems Corporation

Additional Information:	
**Question**	**Response**
**Financial Disclosure**<br><br>Enter a financial disclosure statement that describes the sources of funding for the work included in this submission. Review the submission guidelines for detailed requirements. View published research articles from *PLOS ONE* for specific examples.<br><br>This statement is required for submission and **will appear in the published article** if the submission is accepted. Please make sure it is accurate.<br><br>**Funded studies**<br>Enter a statement with the following details:<br>• Initials of the authors who received each award<br>• Grant numbers awarded to each author<br>• The full name of each funder<br>• URL of each funder website<br>• Did the sponsors or funders play any role in the study design, data collection and analysis, decision to publish, or preparation of the manuscript?<br><br>Did you receive funding for this work?	Yes
Please add funding details.<br>as follow-up to "**Financial Disclosure**<br><br>Enter a financial disclosure statement that describes the sources of funding for the work included in this submission. Review the submission guidelines for detailed requirements. View published research articles from *PLOS ONE* for specific examples.<br><br>This statement is required for submission and **will appear in the published article** if the submission is accepted. Please make sure it is accurate.	This study was supported by the US President's Emergency Plan for AIDS Relief (PEPFAR) through the Centers for Disease Control and Prevention (CDC) under the terms of GGH002520.

**Funded studies**<br>Enter a statement with the following details:<br>• Initials of the authors who received each award<br>• Grant numbers awarded to each author<br>• The full name of each funder<br>• URL of each funder website<br>• Did the sponsors or funders play any role in the study design, data collection and analysis, decision to publish, or preparation of the manuscript?<br><br>Did you receive funding for this work?"	
Please select the country of your main research funder (please select carefully as in some cases this is used in fee calculation).<br>as follow-up to "**Financial Disclosure**<br><br>Enter a financial disclosure statement that describes the sources of funding for the work included in this submission. Review the submission guidelines for detailed requirements. View published research articles from *PLOS ONE* for specific examples.<br><br>This statement is required for submission and **will appear in the published article** if the submission is accepted. Please make sure it is accurate.<br><br>**Funded studies**<br>Enter a statement with the following details:<br>• Initials of the authors who received each award<br>• Grant numbers awarded to each author<br>• The full name of each funder<br>• URL of each funder website<br>• Did the sponsors or funders play any role in the study design, data collection and analysis, decision to publish, or preparation of the manuscript?<br><br>Did you receive funding for this work?"	UNITED STATES - US
**Competing Interests**	All authors declared no competing interests.

Use the instructions below to enter a competing interest statement for this submission. On behalf of all authors, disclose any competing interests that could be perceived to bias this work—acknowledging all financial support and any other relevant financial or non-financial competing interests.<br><br>This statement is required for submission and **will appear in the published article** if the submission is accepted. Please make sure it is accurate and that any funding sources listed in your Funding Information later in the submission form are also declared in your Financial Disclosure statement.<br><br>View published research articles from *PLOS ONE* for specific examples.<br><br>**NO authors have competing interests**<br><br>Enter: *The authors have declared that no competing interests exist.*<br><br>**Authors with competing interests**<br><br>Enter competing interest details beginning with this statement:<br><br>*I have read the journal's policy and the authors of this manuscript have the following competing interests: [insert competing interests here]*<br><br>* typeset	
**Ethics Statement**<br><br>Enter an ethics statement for this submission. This statement is required if the study involved:<br><br>• Human participants<br>• Human specimens or tissue<br>• Vertebrate animals or cephalopods<br>• Vertebrate embryos or tissues	The study was conducted in accordance with the Declaration of Helsinki in accordance to protocol 104/CNBS/2024 approved by the Mozambique National Bioethics Committee for Health. This activity was reviewed by the U.S. Centers for Disease Control and Prevention, deemed research not involving human subjects, and was conducted consistent with applicable federal law and CDC policy. The data used in this analysis are owned by the Ministry of Health Mozambique and come from aggregate data reports, without any personally identifiable information.

• Field research<br><br>Write "N/A" if the submission does not require an ethics statement.<br><br>General guidance is provided below. Consult the submission guidelines for detailed instructions. **Make sure that all information entered here is included in the Methods section of the manuscript.**	

**Format for specific study types**<br><br>**Human Subject Research (involving human participants and/or tissue)**<br>• Give the name of the institutional review board or ethics committee that approved the study<br>• Include the approval number and/or a statement indicating approval of this research<br>• Indicate the form of consent obtained (written/oral) or the reason that consent was not obtained (e.g. the data were analyzed anonymously)<br><br>**Animal Research (involving vertebrate animals, embryos or tissues)**<br>• Provide the name of the Institutional Animal Care and Use Committee (IACUC) or other relevant ethics board that reviewed the study protocol, and indicate whether they approved this research or granted a formal waiver of ethical approval<br>• Include an approval number if one was obtained<br>• If the study involved *non-human primates*, add *additional details* about animal welfare and steps taken to ameliorate suffering<br>• If anesthesia, euthanasia, or any kind of animal sacrifice is part of the study, include briefly which substances and/or methods were applied<br><br>**Field Research**<br><br>Include the following details if this study involves the collection of plant, animal, or other materials from a natural setting:<br>• Field permit number<br>• Name of the institution or relevant body that granted permission	
**Data Availability**<br><br>Authors are required to make all data underlying the findings described fully available, without restriction, and from the time of publication. PLOS allows rare exceptions to address legal and ethical concerns. See the PLOS Data Policy and FAQ for detailed information.	Yes - all data are fully available without restriction

A Data Availability Statement describing where the data can be found is required at submission. Your answers to this question constitute the Data Availability Statement and **will be published in the article**, if accepted.<br><br>**Important:** Stating 'data available on request from the author' is not sufficient. If your data are only available upon request, select 'No' for the first question and explain your exceptional situation in the text box.<br><br>Do the authors confirm that all data underlying the findings described in their manuscript are fully available without restriction?	
**Describe where the data may be found in full sentences. If you are copying our sample text, replace any instances of XXX with the appropriate details.**<br><br>• If the data are **held or will be held in a public repository**, include URLs, accession numbers or DOIs. If this information will only be available after acceptance, indicate this by ticking the box below. For example: *All XXX files are available from the XXX database (accession number(s) XXX, XXX.).*<br>• If the data are all contained **within the manuscript and/or Supporting Information files**, enter the following: *All relevant data are within the manuscript and its Supporting Information files.*<br>• If neither of these applies but you are able to provide **details of access elsewhere**, with or without limitations, please do so. For example:<br><br>*Data cannot be shared publicly because of [XXX]. Data are available from the XXX Institutional Data Access / Ethics Committee (contact via XXX) for researchers who meet the criteria for access to confidential data.*<br><br>*The data underlying the results presented in the study are available from (include the name of the third party*	All relevant data are within the manuscript and its Supporting Information files

and contact information or URL).<br>• This text is appropriate if the data are owned by a third party and authors do not have permission to share the data.<br><br>* typeset	
Additional data availability information:	

Powered by Editorial Manager® and ProduXion Manager® from Aries Systems Corporation

Click here to access/download;Manuscript;Manuscript Data Cleaning - PLOS One v1 13032026_CLEAN.docx

How antiretroviral data cleaning reshaped Mozambique's progress to reaching HIV epidemic control

Aleny Couto[1¶], Orrin Tiberi[1,2¶*], Lindsay Templin[3¶], Morais da Cunha[1], Jose R. Mondlane[1], Jose Mizela[3] , Hermínio Nhaguiombe[3] , Ryan Keating[3] , Ferreira Ferreira[4], Emilton Cumbana[1], Irénio Gaspar[1]

[1.] National Control Program for STIs and HIV/AIDS, National Public Health Directorate, Ministry of Health, Maputo, Mozambique

[2.] Department of Interdisciplinary Social Science: Public Health, Utrecht University; Netherlands

[3.] Division of Global HIV & TB, U.S. Centers for Disease Control and Prevention, Maputo, Mozambique

[4.] U.S. Agency for International Development, Maputo, Mozambique

*affiliation at time of study

*Corresponding author:

E-mail: otiber@gmail.com (OT)

¶These authors contributed equally to this work.

Abstract

Background: Quality data is fundamental for any public health program, especially for long-term care programs that rely on longitudinal data for decision making. In Mozambique, there had been a growing discrepancy between the number of people living with HIV (PLHIV) on antiretroviral treatment (ART) reported in the national system when compared to pharmacy and electronic medical records. A national ART data cleaning exercise was conducted in the first semester of 2024 to improve the accuracy and reliability of the nationally reported data.

Methods: All health facilities with ART services (n=1,754) triangulated their nationally reported data between the clinical data available at the health facility and the pharmacy medication pick-up data. The records that were successfully triangulated were marked as active, while the others were non-active. Upon completion of the review, the nationally reported data were revised.

Results: There was a 10.3% decrease (2,166,941 to 1,944,204) in the number of PLHIV considered currently on ART between December 2023 and June 2024. Among age groups, there was a 28.9% decrease among those less than 10 years of age, 26.6% among those between 10 and 19 years, and 8.4% for those 20 years or more. Cabo Delgado was the province with the largest percentage difference (21.1%, -28,799 PLHIV currently on treatment), while Zambezia had the largest absolute decline, of 41,445 (-8.9%).

Discussion: The decreases in ART coverage resulted in an increase in Mozambique's estimates for new infections, AIDS-related deaths, and vertical transmission. These updates allowed for a more realistic understanding of the HIV epidemic in Mozambique, including highlighting provinces with large data quality issues evidenced by substantial changes in PLHIV on ART numbers. This activity highlighted challenges that exist in paper-based reporting systems and provides evidence for a renewed focus on improving data quality for decision making.

Introduction

The Joint United Nations Programme on HIV/AIDS (UNAIDS) has recognized data as one of the three critical cross-cutting areas to inform national HIV responses [1]. One of the most important indicators for monitoring the performance of HIV programs globally is the number of people living with HIV (PLHIV) currently receiving antiretroviral therapy (ART). This indicator is crucial for the UNAIDS HIV estimation model, Spectrum, which provides data on a country's progress towards achieving epidemic control as defined by the 95-95-95 targets [2,3]. On a yearly basis Mozambique develops and publishes Spectrum estimates that help guide the HIV response in the country. These estimates are developed using multiple inputs, including country programmatic data, national and international surveys, census data, and recent research [4]. Mozambique uses the estimates to inform strategic planning, resource allocation, and program monitoring [3,5,6].

The National Control Program for STIs and HIV/AIDS in Mozambique is supported by the President's Emergency Plan for AIDS Relief (PEPFAR), which provides financial and technical assistance to the Mozambican Ministry of Health (MoH) for the HIV response. With this support, the country has been able to put over two million people living with HIV on ART. Reporting is done through different mechanisms for PEPFAR and MoH. The number of PLHIV on ART is reported to PEPFAR through an electronic medical record (EMR) from approximately 640 health facilities with high ART volume, while the MoH relies on manual counts of paper-based client charts reported to an aggregated electronic system [7].

The manual nature of reporting poses significant challenges to maintaining accuracy across program areas in Mozambique [8–10]. A 2024 national data quality assessment (DQA) revealed that 18% (n=347) of the 1,926 client charts sampled lacked accompanying pharmacy records to confirm ART pick-up [11]. Routine program validations, including DQAs and triangulation of results against other data systems, suggested an increasing misalignment within the reported number of PLHIV on ART. At the end of 2023, there was an 11% difference in the number of PLHIV currently on ART reported between PEPFAR and the MoH, as well

as a 19% difference between the national aggregate pharmacy ART consumption data and the MoH reported results [12,13]. The result of these challenges have already been seen, with data on HIV positivity among pregnant women removed from the UNAIDS estimates process due to continued quality issues [14].

To address this misalignment, the MoH undertook a data cleaning exercise in all health facilities with ART services. In this manuscript, we report on the process and impact of Mozambique's 2024 ART data cleaning exercise. By describing the justification, process and results of the chart review process and the impact of these findings on Mozambique's estimates for tracking progress to HIV epidemic control, we hope that other countries will be encouraged to institute a similar initiative.

Methods

Process of ART data cleaning exercise

The ART data cleaning exercise took place between December 2023 and June 2024 among 1,754 health facilities providing ART services in Mozambique. The HIV program in the MoH provided the orientation and tools for the health facility staff to review every client chart and crosscheck against pharmacy dispensation records to validate a client's current engagement in ART. Following validation, the age of currently engaged clients was updated by data clerks and results were tallied into a revised total of PLHIV currently on ART.

In cases where physical records were missing or unclear, facilities were authorized to consult the individual-level EMR and electronic pharmacy systems where available. Although these electronic systems were not officially used for validation purposes, they were instrumental in locating records, particularly in high-volume health facilities. Once verified, charts were then sorted according to updated status and ART registration number, effectively producing a clean set of active client records.

Data Analysis

To assess the impact of the data cleaning activity, we analyzed the absolute and percent change in the number of PLHIV on ART from results reported in December 2023 (pre-data cleaning) to results reported in June 2024 (post data-cleaning). To evaluate the reliability of the updated reported results, we calculated the change in the percent difference between PEPFAR-reported PLHIV on ART (reported from an EMR) and manually reported PLHIV on ART. PEPFAR defines PLHIV on ART as those currently receiving ART according to the national treatment protocol, not counting those who have not received ARVs ≤28 days from their last expected pickup, while the manual reported data uses a ≤59-day threshold [5]. Alignment of the reported results between the systems is considered reliable if the difference is less than 5%, a standard based on client-level analyses of the definitional differences [15]. Where possible, comparisons of interest were disaggregated by age (<10, 10-19, and 20+ years), sex (male and female), province, facility volume (1-500, 501-1000, 1000+ PLHIV on ART) and the presence of an EMR at the facility. Data access to the health facility level aggregated datasets at both MoH and PEPFAR occurred between the 23rd and 27th of June 2025. Due to the aggregated nature of these datasets, no personal identifiers were accessed.

HIV Estimates

The internal processes of the Spectrum software package, mathematical modeling tools used to generate HIV estimates, have been extensively described in the literature [3]. Spectrum outputs include estimates for the number of PLHIV, the 95-95-95 cascade, new HIV infections, and HIV-related deaths, as well as future projections. One of the essential inputs for the model is the number of PLHIV on ART. As the data errors in the archive were assumed to have accumulated over time, historical provincial ART data were calculated between 2020, which was the last time there was a national recount of the HIV archive, and the updated 2024 PLHIV on ART. The start year was also based off a recent analysis which found the absolute different in 2020 between PLHIV currently on ART between PEPFAR and MoH was only 0.10%, or 1,598 individuals [16]. The yearly distribution weights were based off new ART initiations for 2021 (28% of the total increase), 2022 (28%), and 2023 (25%), after which these new historical treatment data were included in the 2024 Spectrum estimates. These adjusted 2024 estimates were then compared

to the 2024 projections from the previous year's estimates to determine the impact of the ART archive cleaning on the number of PLHIV, the 95-95-95 cascade, new infections, and deaths.

Human ethics and consent to participate

The study was conducted in accordance with the Declaration of Helsinki in accordance to protocol 104/CNBS/2024 approved by the Mozambique National Bioethics Committee for Health. This activity was reviewed by the U.S. Centers for Disease Control and Prevention, deemed research not involving human subjects, and was conducted consistent with applicable federal law and CDC policy. The data used in this analysis are owned by the Ministry of Health Mozambique and come from routine aggregate data reports, without any personally identifiable information.

Results

Results in the MoH reported data

At the conclusion of the data cleaning exercise, there was a 10.3% (n=222,737) reduction in the number of PLHIV on ART between December 2023 (2,166,941) and June 2024 (1,944,204) in the national reporting system. A decrease of 28.9% (23,686) was seen among children aged 0-10 years, 26.6% for adolescents 10-19 years (35,729), and 8.4% (163,322) among adults aged ≥20 years. (Table 1) When disaggregated by sex among adolescents, the percentage difference was closely aligned between female (26.4%) and males (26.9%). However, there is almost double the difference was nearly double among adults aged ≥20 years by sex; females decreased 7.0% and males 11.0%.

Table 1: Comparison of number of people with HIV currently on antiretroviral therapy (ART) before and after the 2024 data cleaning exercise, Mozambique (n=1754 healthcare facilities)

		Pre-Data Cleaning (December 2023)	Post-Data Cleaning (June 2024)	Change (Pre-Post)	Percent Change
**Total**		**2,166,941**	**1,944,204**	**- 222,737**	**-10.3%**
**Reported Sex and Age Groups**	**<10 years**	81,817	58,131	- 23,686	-28.9%
	**10-19 years**	134,228	98,499	- 35,729	-26.6%
	**Female 10-19 years**	84,783	62,369	- 22,414	-26.4%
	**Male 10-19 years**	49,445	36,130	- 13,315	-26.9%
	**≥20 years**	1,950,896	1,787,574	- 163,322	-8.4%
	**Female ≥20 years**	1,273,586	1,184,689	- 88,897	-7.0%
	**Male ≥20 years**	677,310	602,885	- 74,425	-11.0%
**Province**	**Niassa**	66,397	55,829	- 10,568	-15.9%
	**Cabo Delgado**	136,670	107,891	- 28,779	-21.1%
	**Nampula**	290,745	249,488	- 41,257	-14.2%
	**Zambezia**	464,170	422,725	- 41,445	-8.9%
	**Tete**	127,268	117,077	- 10,191	-8.0%
	**Manica**	160,824	137,713	- 23,111	-14.4%
	**Sofala**	204,413	171,127	- 33,286	-16.3%
	**Inhambane**	117,585	114,295	- 3,290	-2.8%
	**Gaza**	216,497	211,931	- 4,566	-2.1%
	**Maputo Province**	206,004	185,967	- 20,037	-9.7%
	**Maputo City**	176,368	170,161	- 6,207	-3.5%

**Facility PLHIV on ART Volume**	**Low Volume (1-500)**	178,495	177,254	- 1,241	-0.7%
	**Medium Volume (501-1,000)**	193,020	154,082	- 38,938	-20.2%
	**High Volume (>1,000)**	1,795,426	1,612,868	- 182,558	-10.2%
**Presence of Electronic Medical Record at Facility**	**No**	377,058	313,325	- 63,733	-16.9%
	**Yes**	1,789,883	1,630,879	- 159,013	-8.9%

Results between MoH data and PEPFAR at health facilities with EMR

At health facilities with an EMR, the post-review results indicate a greater alignment between the two reporting systems for PLHIV currently on ART, dropping from a 6.5% difference in December 2023 to a difference of 1.0% in June 2024 (Table 2). Throughout the review, PEPFAR's count of PLHIV on ART decreased from 1,676,529 to 1,614,096, or a 1.9% decline, while the MoH's count of PLHIV on ART in sites with EMR fell from 1,789,883 to 1,630,870 representing a 4.6% decrease. The decreases in these data were mitigated by the identification and enrollment of out-of-care PLHIV during the same period through established programs of the HIV program. In the first six months of 2024 there were 136,256 newly initiated PLHIV on ART, and an additional 85,533 PLHIV who returned to treatment after a documented care

interruption [13]. Without these additional entries into ART services, the absolute decrease in PLHIV on ART would have been substantially more.

Table 2: Comparison between PEPFAR and Ministry of Health reports of the number of people with HIV (PLHIV) currently on antiretroviral treatment (ART) in Electronic Medical Records (EMR) sites before and after the 2024 Data Cleaning, Mozambique (n=640 healthcare facilities)

		PLHIV on ART: PEPFAR vs MoH (Pre-Data Cleaning December 2023)				PLHIV on ART: PEPFAR vs MoH (Post-Data Cleaning June 2024)			
		PEPFAR PLHIV on ART	MoH PLHIV on ART	Abs. Difference	Percent Difference	PEPFAR PLHIV on ART	MoH PLHIV on ART	Abs. Difference	Percent Difference
**Total**		**1,676,529**	**1,789,883**	**113,354**	**6.5%**	**1,614,096**	**1,630,879**	**16,783**	**1.0%**
**Reported Age Groups**	**<10 years**	46,289	65,744	19,455	34.7%	41,805	47,821	6,016	13.4%
	**10-19 years**	67,334	106,476	39,142	45.0%	63,859	79,131	15,272	21.4%
	**Female 10-19**	43,042	68,488	25,446	45.6%	39,649	50,738	11,089	24.5%
	**Male 10-19**	24,292	37,988	13,696	44.0%	24,210	28,393	4,183	15.9%
	**≥20 years**	1,562,906	1,617,663	54,757	3.4%	1,508,432	1,503,927	4,505	0.3%
	**Female ≥20 years**	1,047,683	1,053,536	5,853	0.6%	1,015,299	993,742	21,557	2.1%
	**Male ≥20 years**	515, 223	564,127	48,904	8.7%	493,133	510,185	17,052	3.4%
**Province**	**Niassa**	33,699	37,538	3,839	10.8%	33,814	34,939	1,125	3.3%
	**Cabo Delgado**	82,467	93,179	10,712	12.2%	75,569	80,338	4,769	6.1%
	**Nampula**	198,446	221,016	22,570	10.8%	173,971	192,357	18,386	10.0%
	**Zambezia**	375,401	405,866	30,465	7.8%	382,375	374,914	7,461	2.0%

	**Tete**	95,300	97,453	2,153	2.2%	95,811	92,455	3,356	3.6%
	**Manica**	133,926	140,145	6,219	4.5%	121,443	121,163	280	0.2%
	**Sofala**	147,554	150,030	2,476	1.7%	128,163	126,538	1,625	1.3%
	**Inhambane**	82,403	83,956	1,553	1.9%	84,740	82,042	2,698	3.2%
	**Gaza**	178,337	185,719	7,382	4.1%	181,310	181,635	325	0.2%
	**Maputo Province**	165,486	183,614	18,128	10.4%	152,880	159,723	6,843	4.4%
	**Maputo City**	150,313	157,663	7,350	4.8%	151,950	151,925	25	0.0%
	**Military**	33,197	33,704	507	1.5%	32,070	32,850	780	2.4%
**Facility PLHIV on ART Volume**	**Low Volume (1-500)**	14,595	15,076	481	3.2%	14,844	15,185	341	2.3%
	**Medium Volume (501-1,000)**	81,394	84,427	3,033	3.7%	85,334	82,445	2,889	3.4%
	**High Volume (>1,000)**	1,580,540	1,690,380	109,840	6.7%	1,513,918	1,533,249	19,331	1.3%

Focusing specifically on adolescents aged 10-19 years, the data reveal a discrepancy between the figures reported by PEPFAR and the MoH in December 2023, with a difference of 45.0% (67,334 for PEPFAR and 106,476 for MoH). During the subsequent exercise this difference decreased to 21.4% in June 2024 (absolute difference of 15,272). The number of adolescent PLHIV reported to PEPFAR decreased 2.6% from 67,334 to 63,859, while the MoH saw a more substantial decline of 14.7% (106,476 to 79,131) in these sites.

Results in the National Estimates Projection

As a result of the data cleaning exercise, national ART coverage among all PLHIV dropped from 89% to 87%, with the largest drop among children 0-14 years (79% to 55%), in the 2024 estimates (Table 3). Overall new HIV infections increased by 22.7% (17,000), AIDS-related deaths rose by 12.8% (5,000), and vertical transmission rates increased from 8.5% to 12.3%. Children 0-14 years emerged with the largest relative changes in the estimates. The updated HIV estimates show a 28.6% (40,000) increase in the number of children living with HIV, a 70.0% (7,000) rise in new infections and a 49.3% (3,300) increase in AIDS-related deaths.

Table 3: Comparison of results between the 2024 projections and the 2024 estimates for selected indicators.

Year: 2024	Projections (Upper-Lower Bounds)	Estimates (Upper-Lower Bounds)	Change (Pre-Post)	Percent Change
HIV Prevalence (15-49 years):	**11.08** [10.52 - 11.68]	**11.46** [10.83 - 12.12]	0.38	3.4%
Vertical Transmission (%)	**8.5** [7.8 – 9.7]	**12.3** [9.7 - 14.4]	3.8	32.3%
People living with HIV on ART	2 220 000	2 050 000	170,000	7.7%

**ART Coverage:**[a]	**88.7%**	**81.8%**	**-6.9%**	**-7.8%**
Children (0-14 years):	**78.8%**	**54.6%**	**-24.2%**	**-30.7%**
Women (15+ years):	**91.7%**	**85.1%**	**-6.6%**	**-7.2%**
Pregnant Women (15+ years)[b]	***91.4%***	***81.7%***	***-9.7%***	***-10.6%***
Men (15+ years):	**85.2%**	**81.7%**	**-3.5%**	**-4.1%**
**People living with HIV:**[a]	**2 500 000** [2 300 000 - 2 700 000]	**2 500 000** [2 300 000 - 2 700 000]	**0**	**0.0%**
Children (0-14 years):	**140 000** [120 000 - 160 000]	**180 000** [150 000 - 200 000]	**40,000**	**28.6%**
Adolescents (10-19 years)[b]	***140 000*** *[100 000 - 170 000]*	***160 000*** *[110 000 - 200 000]*	**20,000**	**14.3%**
Women (15+ years):	**1 500 000** [1 400 000 - 1 600 000]	**1 500 000** [1 400 000 - 1 700 000]	**0**	**0.0%**
Men (15+ years):	**830 000** [770 000 - 910 000]	**800 000** [730 000 - 880 000]	**-30,000**	**-3.6%**
**New Infections:**[a]	**75 000** [59 000 - 94 000]	**92 000** [73 000 - 114 000]	**17,000**	**22.7%**
Children (0-14 years):	**10 000** [9 000 - 12 000]	**17 000** [11 000 - 21 000]	**7,000**	**70.0%**
Adolescents (10-19 years)[b]	***14 000*** *[3 000 - 39 000]*	***17 000*** *[3 000 - 29 000]*	**3,000**	**21.4%**
Women (15+ years):	**42 000** [32 000 - 53 000]	**49 000** [38 000 - 63 000]	**7,000**	**16.7%**
Men (15+ years):	**23 000** [17 000 – 29 000]	**26 000** [20 000 – 34 000]	**3,000**	**13.0%**
**Deaths:**[a]	**39 000** [34 000 - 46 000]	**44 000** [37 000 - 52 000]	**5,000**	**12.8%**
Children (0-14 years):	**6 700** [4 700 - 8 500]	**10 000** [6 700 – 13 000]	**3,300**	**49.3%**
Adolescents (10-19 years)[b]	***2 500*** *[1 700 - 3 400]*	***3 100*** *[2 300 - 4 100]*	**600**	**24.0%**
Women (15+ years):	**17 000** [15 000 - 21 000]	**19 000** [16 000 - 23 800]	**2,000**	**11.8%**
Men (15+ years):	**15 000** [12 000 - 18 000]	**15 000** [12 000 – 17 000]	**0**	**0.0%**

[a] Due to rounding, the disaggregation of the total may not always sum to the total

[b] Adolescents and Pregnant Women are shown as separate groups; however, they overlap with other age/sex categories

NOTE: The projections were elaborated in 2023 with pre-cleaning data, while the estimates were elaborated in 2024 and use the post-cleaning data

Discussion

The ART data cleaning exercise, carried out between June 2023 and December 2024, demonstrated measurable improvements in the accuracy and consistency of Mozambique's national ART data. Within a 12-month period, the difference between the PEPFAR EMR-based reported data and the MoH paper-based reported data decreased from 6.5% to 1.0%. Led by the MoH and implemented by provincial teams, the process revealed discrepancies across reporting systems, providing critical insights into data management and client monitoring challenges that can inform future health information strengthening efforts. While the 11.5% decrease in ART coverage may appear as a setback, the long-term benefits of accurate, reliable data far outweigh these temporary challenges.

The chart review revealed considerable discrepancies between previously reported figures and revised data, exposing long-standing challenges in client monitoring, data management, and reporting systems. Paper-based records continue to undermine the integrity of data across the entire lifecycle. Data fragmentation and duplication are also pressing issues, often arising from unlinked health facility records or parallel reporting systems. Although initiatives to improve data systems are underway, progress is uneven and implementing a systemwide review may be beneficial for a unified national approach.

These adjustments have had a ripple effect on national Spectrum HIV estimates. The increase in new infections aligns with the lower ART coverage in the updated estimates, showing the impact of a higher number of undiagnosed and untreated individuals than previously estimated [17]. Increases in AIDS-related deaths may reflect a more accurate picture of mortality among PLHIV, including the higher risk of mortality

among PLHIV with advanced HIV disease [18,19]. While increases in rates of vertical transmission point to continued challenges, especially in the coverage of prevention of mother-to-child transmission efforts among all HIV-positive pregnant women [20]. Among all demographic groups, the increases in estimates for adolescents living with HIV underscore the urgency of strengthening effective adolescent-focused interventions, including efforts to improve low ART coverage such as improving early diagnosis and ensuring long-term care retention for this priority population [21].

This exercise came at a time when the country committed to the achievement of the 95-95-95 targets by the end of 2025. Prior to the data cleaning in 2023, the national HIV care cascade was reported at 89-97-90 [8]. However, at the end of 2024 the cascade now reflects a more realistic but lower coverage rate of 87-94-91, directly influenced by the reduction in reported ART clients [13]. The adolescent-specific cascade presents an even graver situation, standing currently at 70-96-77. This highlights substantial gaps in adolescent HIV testing and retention, necessitating focused corrective measures. The updated cascades allow the country to have a more realistic understanding of the state of the HIV response and to set priorities for the coming years to ensure progress toward epidemic control and eventually ending HIV as a public health threat by 2030.

Reflections

Even after the review, many of the challenges identified in the cleaning exercise persist. Recognizing the value of the data cleaning initiative, the MoH has recommended that this activity be institutionalized as a routine annual process, with structured oversight from district and provincial health authorities [22]. Institutionalizing targeted routine data cleaning exercises can yield several benefits, including up-to-date and accurate client records and more realistic national targets aligned with service delivery and population needs. These improvements are critical to a successfully coordinated national response and ultimately can improve the country's ability to eliminate HIV as a public health threat. Routine data quality interventions, along with broader investments in health information systems, are core elements of national efforts to

achieve HIV epidemic control. Achieving these outcomes requires investment in data quality, including human resource capacity, digital health infrastructure, and stronger monitoring and evaluation frameworks with clear indicators, regular supervision, and accountability mechanisms.

Limitations

There are some important limitations to consider from this exercise. Limited resources prevented supervision visits to all facilities and data cleaning validations focused on larger, more centrally located health facilities. In contrast, rural and smaller health facilities without donor support, which make up most of health facilities with ART services, did not have regular supervision visits for validation despite recording the largest differences in the analysis. The results presented for these health facilities may be larger or smaller than reported depending on implementation fidelity, especially in provinces such as Inhambane and Gaza where the EMR was used for reporting cleaning data. In addition, data reporting in the national system can vary depending on many factors such as reporting error, climatic events and social unrest. Some discrepancies in reported PLHIV on ART from both before and after the exercise may have been impacted by these factors.

Conclusions

The 2024 ART data cleaning exercise provided a more accurate foundation for monitoring Mozambique’s HIV response and highlights the importance of data quality, triangulation, and accuracy. Despite the discouraging impact on the HIV estimates, the Government of Mozambique recognized that more accurate data were essential for effective planning, prioritization, and accountability. The cleaning exercise created a more reliable baseline for program performance and target-setting, while exposing systemic weaknesses in the health information system previously obscured by incorrect data. In this sense, the chart review exercise played a pivotal role not only in revealing the true scope of the HIV epidemic but also providing the rationale towards increased focus on improving quality for data-driven decision-making.

Acknowledgements

This activity would not have been possible without the collaboration of Isabel Sathane, Helio Mabjaia, Stelio Maposse, and Makini Boothe. The support of the provincial HIV program staff was also indispensable. In particular we would like to thank the provinces of Sofala, especially Aquinoa Matlonhana, Dr. Justina Zucule, and Giuseppe Garfo, and Zambézia, especially Remígio Chicuava and Dr. Odilho Sande.

Author contributions

Conceptualization: Aleny Couto, Orrin Tiberi, Lindsay Templin, Morais da Cunha, Jose R. Mondlane

Data curation: Lindsay Templin, Orrin Tiberi

Formal analysis: Herminio Nhaguiombe, Jose Mizela, Lindsay Templin, Orrin Tiberi

Investigation: Lindsay Templin, Orrin Tiberi

Methodology: Morais da Cunha, Aleny Couto, Irénio Gaspar, Orrin Tiberi

Resources: Aleny Couto, Ryan Keating, Irénio Gaspar

Supervision: Aleny Couto, Ryan Keating

Validation: Orrin Tiberi, Lindsay Templin

Visualization: Orrin Tiberi

Writing – original draft: Aleny Couto, Lindsay Templin, Orrin Tiberi

Writing – review & editing: Aleny Couto, Orrin Tiberi, Lindsay Templin, Jose R Mondlane, Morais da Cunha, Jose Mizela, Herminio Nhaguiombe, Ryan Keating, Ferreira Ferreira, Emilton Cumbana

References

1. UNAIDS. Global AIDS Strategy 2021-2026 End Inequalities. End AIDS. Executive Summary. Geneva, Switzerland: UNAIDS; 2021.

2. UNAIDS. Political Declaration on HIV and AIDS: Ending Inequalities and Getting on Track to End AIDS by 2030. Geneva, Switzerland: UNAIDS; 2021.

3. van Schalkwyk C, Mahy M, Johnson LF, Imai-Eaton JW. Updated Data and Methods for the 2023 UNAIDS HIV Estimates. JAIDS J Acquir Immune Defic Syndr. 2024;95. Available: https://journals.lww.com/jaids/fulltext/2024/01011/updated_data_and_methods_for_the_2023_unaids_hiv.1.aspx

4. van Schalkwyk C, Mahy M, Johnson LF, Imai-Eaton JW. Updated Data and Methods for the 2023 UNAIDS HIV Estimates. JAIDS J Acquir Immune Defic Syndr. 2024;95: e1–e4. doi:10.1097/qai.0000000000003344

5. PEPFAR. Monitoring, Evaluation, and Reporting Indicator Reference Guide 2.8.2. Washington D.C: PEPFAR; 2025 Mar.

6. UNAIDS. Spectrum v6.42. 2024 [cited 28 June 2025]. Available: https://hivtools.unaids.org/spectrum-file-request/

7. Guião de cuidados do HIV do Adulto, Adolescente Grávida, Lactante e Criança (HIV Care Guide for Adults, Pregnant Adolescents, Lactating Women and Children). Maputo, Mozambique: Programa Nacional de Controle de ITS e HIV/SIDA, Ministério de Saúde; 2023.

8. Relatório Anual de HIV, 2023 (Annual HIV Report). Mozambique: Programa Nacional de Controle de ITS e HIV/SIDA, Ministério de Saúde; 2023.

9. Colborn JM, Zulliger R, Da Silva M, Mathe G, Chico AR, Castel-Branco AC, et al. Quality of malaria data in public health facilities in three provinces of Mozambique. PLOS ONE. 2020;15: e0231358. doi:10.1371/journal.pone.0231358

10. Sambo J, Chicumbe S, de Deus N, Gonçalves L. Quality of routine data for monitoring nutrition and diarrhoea indicators of children under 5 in Mozambique: an ecological study over a 5-year period. BMJ Open. 2023;13: e073239. doi:10.1136/bmjopen-2023-073239

11. Direcção Nacional de Saúde Pública. Relatório da Ronda Nacional da Avaliação da Qualidade de Dados 2024 (National Data Quality Assessment Report 2024). Maputo, Mozambique: Programa Nacional de Controle de ITS e HIV/SIDA, Ministério de Saúde; 2024.

12. Direcção Nacional de Saúde Pública. Relatório da Ronda Nacional da Avaliação da Qualidade de Dados 2023 (National Data Quality Assessment Report 2023). Maputo, Mozambique: Programa Nacional de Controle de ITS e HIV/SIDA, Ministério de Saúde; 2023.

13. Relatório Anual de HIV, 2024 (Annual HIV Report). Mozambique: Programa Nacional de Controle de ITS e HIV/SIDA, Ministério de Saúde; 2024.

14. Stevens O, Boothe M, Tiberi O, Mahy M, Walker P, Glaubius R, et al. Triangulation of Routine Antenatal HIV Prevalence Data and Adjusted HIV Estimates in Mozambique. JAIDS J Acquir Immune Defic Syndr. 2024;95. Available: https://journals.lww.com/jaids/fulltext/2024/01011/triangulation_of_routine_antenatal_hiv_prevalence.8.aspx

15. Relatório Trimestral do MozART (Quarterly MozART Report). Maputo, Mozambique; Centers for Disease Control and Prevention; 2024.

16. Tiberi O, Templin L, Ferreira F, Nhaguiombe H, Couto A, Lara J, et al. Alinhamento de Dados Entre os Sistemas de Reporte de HIV em Moçambique, 2017-2023. Rev Mocambicana Cienc Saude. 2024;10: 23–26.

17. Cohen MS, Chen YQ, McCauley M, Gamble T, Hosseinipour MC, Kumarasamy N, et al. Antiretroviral Therapy for the Prevention of HIV-1 Transmission. N Engl J Med. 2016;375: 830–839. doi:10.1056/nejmoa1600693

18. Glaubius R, Kothegal N, Birhanu S, Jonnalagadda S, Mahiane SG, Johnson LF, et al. Disease progression and mortality with untreated HIV infection: evidence synthesis of HIV seroconverter cohorts, antiretroviral treatment clinical cohorts and population-based survey data. J Int AIDS Soc. 2021;24: e25784. doi:10.1002/jia2.25784

19. Portilla-Tamarit J, Reus S, Portilla I, Fuster Ruiz-de-Apodaca MJ, Portilla J. Impact of Advanced HIV Disease on Quality of Life and Mortality in the Era of Combined Antiretroviral Treatment. J Clin Med. 2021;10. doi:10.3390/jcm10040716

20. Walters MK, Bulterys M, Barry M, Louden D, Hicks S, Richey A, et al. Probability of vertical HIV transmission: A systematic review and meta-regression. medRxiv : the preprint server for health sciences. United States; 2024. p. 2024.12.03.24318418. doi:10.1101/2024.12.03.24318418

21. Tiberi O, McCabe KC, Baltazar CS, Boothe M, de Deus MI, Ribeiro E, et al. Prevalence and determinants of being out-of-care among HIV-positive young people aged 15–24 years in Mozambique: findings from the 2021 population-based HIV impact assessment survey. BMC Infect Dis. 2026;26: 460. doi:10.1186/s12879-026-12745-5

22. Direcção Nacional de Saúde Pública. Relatório da Limpeza de Dados dos Serviços TARV, 2024 (Report on Data Cleaning in ART Services, 2024). Maputo, Mozambique: Programa Nacional de Controle de ITS e HIV/SIDA, Ministério de Saúde; 2024.

Raw Data

Click here to access/download
Supporting Information - Compressed/ZIP File Archive
Data for PLOS One.zip
