# Supplementary material for: How antiretroviral data cleaning reshaped Mozambique’s progress to reaching HIV epidemic control
Source: PLoS One. 2026 Jul 29;21(7):e0354368. doi: 10.1371/journal.pone.0354368 (PMC13419232; doi:10.1371/journal.pone.0354368)
Supplement: Attachment — Submitted filename: PONE Round 1 Reviewer Resposes 12052026.pdf [file pone.0354368.s004.pdf]

13/May/2026

Dear PLOS One Editors,

Thank you for your review, please find your questions/comments and the following responses highlighted in **bold/green**.

With Regards,

Orrin Tiberi

General Feedback

1.Please ensure that your manuscript meets PLOS ONE's style requirements, including those for file naming. The PLOS ONE style templates can be found at

https://journals.plos.org/plosone/s/file?id=wjVg/PLOSOne_formatting_sample_main_body.pdf and

https://journals.plos.org/plosone/s/file?id=ba62/PLOSOne_formatting_sample_title_authors_affiliations.pdf

Thank you, I have updated what I have found to be out of alignment with the style requirements.

2. Thank you for stating the following financial disclosure:

"This study was supported by the US President's Emergency Plan for AIDS Relief (PEPFAR) through the Centers for Disease Control and Prevention (CDC) under the terms of GGH002520."

Please state what role the funders took in the study. If the funders had no role, please state: "The funders had no role in study design, data collection and analysis, decision to publish, or preparation of the manuscript." If this statement is not correct you must amend it as needed.

Please include this amended Role of Funder statement in your cover letter; we will change the online submission form on your behalf.

Updated in the Cover Letter, thank you for the comment and for updating the submission form on my behalf.

3. Please include captions for your Supporting Information files at the end of your manuscript, and update any in-text citations to match accordingly. Please see our Supporting Information guidelines for more information: http://journals.plos.org/plosone/s/supporting-information.

Thank you for the comment, the supporting information is based on the requirements for PLOS One submission (data, human subjects checklist, protocol, and ethical approval letters), so there is no in-text citation. We have provided a list of this supporting information at the end of the manuscript.

4. Thank you for providing your underlying data as Supporting Information.

We note that the data set contains text or data that is not in English. Please note that PLOS is an English-language publisher, so we require data sets to be provided in English as well. Please

upload an English-language version of your data set. **Thank you for the comment, this has been updated.**

This will also allow us to determine if your data follows PLOS standards per our Data Availability policy here: https://journals.plos.org/plosone/s/data-availability

5. If the reviewer comments include a recommendation to cite specific previously published works, please review and evaluate these publications to determine whether they are relevant and should be cited. There is no requirement to cite these works unless the editor has indicated otherwise. **Noted, thank you.**

6. Please review your reference list to ensure that it is complete and correct. If you have cited papers that have been retracted, please include the rationale for doing so in the manuscript text, or remove these references and replace them with relevant current references. Any changes to the reference list should be mentioned in the rebuttal letter that accompanies your revised manuscript. If you need to cite a retracted article, indicate the article's retracted status in the References list and also include a citation and full reference for the retraction notice. – **Thank you for the comment, there were a few reference artifacts from a previous version that created duplication in the references (two copies of Updated Data and Methods for the 2023 UNAIDS HIV Estimates, and an additional reference which was not relevant to the updated text (Guião de cuidados do HIV do Adulto, Adolescente Grávida, Lactante e Criança (HIV Care Guide for Adults, Pregnant Adolescents, Lactating Women and Children)). These have both been corrected.**

[Note: HTML markup is below. Please do not edit.]

5. Review Comments to the Author

Please use the space provided to explain your answers to the questions above. You may also include additional comments for the author, including concerns about dual publication, research ethics, or publication ethics. (Please upload your review as an attachment if it exceeds 20,000 characters)

Reviewer #1: The authors have done well to document a commonly observed and topical phenomenon in ART programs in sub-Saharan Africa. The methods have been well elaborated, and the findings well discussed. The authors' reflections provide further insight into the implications of the findings for practice and policy. However, I have the following recommendations to further increase the understanding and improve reproducibility of the study.
INTRODUCTION
Line 50: Provide one sentence on what the 95-95-95 targets are to improve ease of understanding for persons not previously familiar with the HIV program. **– updated, thank you**
METHODS
Line 78: Replace the word "among" with "across" **– updated, thank you**

Lines 81-82: Please clarify whether the clients' current age computed, or the age as documented in the clinical records as of the time of enrollment into ART were captured? **– updated, thank you, it is calculated from DOB.**

DATA ANALYSIS

Line 96: Was the <=59 days threshold based on the last expected pickup visit or the last documented pickup visit? Please be explicit about the criteria. **– updated, thank you, is from the expected pickup date**

HIV ESTIMATES

Line 111: Replace the word "different" with "difference". **– updated, thank you**

RESULTS

Line 131: Tautology, please revise sentence. **– updated, thank you**

Table 1 - In your reflections, please suggest that future studies may explore what informed the differences in the error rates across provinces. One would have expected provinces with higher client loads to have higher error rates. But this is not established with the results. Perhaps a qualitative study or data management process evaluation could unravel further insight.- **thank you, this has been added to the discussion (lines 162-163) as the reflection is more focused on the ministry recommendations that came out of the exercise.**

Table 1 - You may also explore using some inferential statistics like T-test or Mann Whitney U test depending on the data distribution to establish the nuanced differences in error rates by age, sex, provinces, facility client load, and presence of EMR. **– after discussion with the manuscript authors, we feel that the purpose of these tables are not to measure nuanced differences or changes, but to present a direct picture of the overall impact of data cleaning on the registered PLHIV on ART. As such, we believe that the simpler approach presented in the table is the best method for showing the results, without any additional statistical analysis.**

Lines 140-142: In your reflections, please provide practical recommendations for unifying the reporting system so that there is one single source of truth for PEPFAR and MOH data going forward? **– Thank you for the comment, we have added in a part about the possibility of aligning data reporting between PEPFAR and MoH, however as this issue is quite political at the moment with the USG MOUs, the authors don't feel it is appropriate for their position within the ministry to add additional information.**

Table 2 - In your discussions or reflections please expatiate on why there are still differences in PLHIV on ART between PEPFAR and MOH records post-data cleaning. One would have expected the same cleaned data to feed both information systems. This was not very clear in your methodology section also, so it raises questions in when this is presented in Table 2 results. – **Thank you for the comment, this is included in the methods – data analysis when defining the differences between PEPFAR (≤28 days) and MoH (≤59 days) for PLHIV active on treatment. The same data is used in both systems, however as there is a difference in definition there will never be complete alignment.**

Reviewer #2: This is an important report for Mozambique as well as for many other global settings with high HIV burden. Here, the authors quantify the discrepancy in the number of PLHIV between PEPFAR counts and the Ministry of Health (MOH) in Mozambique before and after a data cleaning exercise. The "before" comparison confirms the important discrepancy. The "after" comparison shows a reduction in discrepancy (an improvement but with persistent discrepancies) as well as important implications in the model-based forecast.

Few issues:

1. In the abstract and the methods, I would suggest reformulating the first sentence in the methods to avoid the word "triangulated". At this stage, not many readers understand that triangulation involves using multiple data sources to verify accuracy and consistency. In fact, in the main text, there is an overuse of the word "triangulation" without ever presenting a definition or some sort of specification in this particular study/analysis. - **– thank you for the comment, we have updated the abstract and defined triangulation in the second to last paragraph of the introduction "an in-depth comparison of individual level records against other data systems" (lines XXXX)**

Also, I would suggest removing the mention of ART coverage in the discussion (in the abstract). Put the number of people living with HIV under ART. **– thank you for the comment, we have updated**

2. The introduction is well written and makes a good case of why it matters to do this study. Lines 55/58 - Please do revise in light of the events last year. - **– thank you for the comment, at the time of the date cleaning activity the USG program was still known as PEPFAR, so we would prefer to keep it true to the time-frame.**

Line 61 - Please confirm citation 7 for the number of health facilities of high-volume or how the MoH aggregates the data. This is because citation 7 seems to be a clinical guide. **– thank you for the comment, this paragraph and the previous one had some citation artifacts from previous versions which have now been updated.**

3. Methods

- Please set a sort of definition of what triangulation means in this work **– thank you for the comment, we have updated the abstract and defined triangulation in the second to last paragraph of the introduction (lines 75-76)**

- It would be helpful to have a figure describing how the triangulation (checking, validation, confirmation, etc.) algorithm is done. **– thank you, we have defined triangulation in the second to last paragraph of the introduction "an in-depth comparison of individual level records against other data systems" (lines 75). The specific triangulation done for this analysis is described in detailed the methods section, line 89 -99**

- Data analysis: Two things about the age. i) What age is used here? Is this age at ART registration or age at the time of the survey, or another sort of age? Please specify. **– updated, thank you, it is calculated from DOB as specificized in line 93**

ii) Age group divisions. It would be good if the age groups are under-5, 5 to 9, 10 to 14, 15 to 19, 20 to 35, and 35 or more. If not possible, please give a justification for the groups chosen. **– thank you for the comment. These age groups were prioritized in the data cleaning (children, adolescents, and adults) and additional the MoH reported data only has 20+, meaning we are also limited by that report. Have clarified this on line 110-111**

- HIV Estimates: Perhaps the title of this subsection should be "HIV forecast estimates". Or choose a title that indicates that some mathematical modeling is used. **– thank you for the comment, we have updated it to read HIV Spectrum Estimates, which is known as a modelling software.**

- HIV Estimates: Lines 110/115 - please rephrase to make it clearer. What are the weights used for? And how were they chosen? **– thank for the comment, please see the updated text on line 123-125**

- About the absolute differences: The absolute differences are a good indicator. But they focus only on the magnitude of the difference and ignore its direction. It matters here to know both. – **thank you for the comment. In general we present the directionality of the absolute difference when it concerns the results from this analysis, however this is not done for background information as we deemed it not necessary. We used absolute difference for the comparison of PEPFAR and SIS-MA as this is a percent difference comparison where there is no implication of one value being the reference or "original" value. We agree that a directionality is helpful but this is technically not sound for this specific comparison. The percent change is calculated in table 1 to show the directionality of the change within SIS-MA from December 2023 to June 2024.**

- About the relative differences: Please indicate how the relative differences are computed. In this, it should be clear which reference (MoH or PEPFAR counts) is used and whether it can be negative or positive.

Thank you for this feedback. We have added the respective calculations as footnotes in the manuscript.

4. For the results:

- It would be good to have a frequency table with the reasons why records were removed or added. What did the "triangulation" lead to 222,737 fewer observations? **– thank you for the comment, this is addressed in the first paragraph of the methods section (lines 89-94) in which we describe the initial crosscheck between patient charts and the pharmacy record and additional verification with the EMR when necessary. We didn't track individual reasons for removal, however as these are were the only steps done it can be assumed that the vast majority of the patient charts removed were due to non-active status.**

- Table 1 - Indicate in a footnote how the percent change is calculated. **Updated at the end of each of the tables, thank you.**

- Table 1: Is it possible to repeat this analysis stratified by urban/rural health facilities? **– thank you for the comment. Unfortunately, we don't have complete data on the rural/urban divide so have decided to leave it out as to not present different totals than the rest of the document. The last information we have to categorize rural/urban is from the 2018 SARA (Service Availability and Readiness Assessment) after which there has been a large expansion of HF in the country.**

- Table 2: We need rural/urban analysis as well. Please indicate in the footnotes how the relative differences are computed. **– thank you for the comment. Unfortunately, we don't have complete data on the rural/urban divide so have decided to leave it out as to not present different totals than the rest of the document. The last information we have to categorize rural/urban is from the 2018 SARA (Service Availability and Readiness Assessment) after which there has been a large expansion of HF in the country.**

- Table 3: Please indicate that these are Spectrum estimates **– updated in the title, thank you**
