# Supplementary material for: How antiretroviral data cleaning reshaped Mozambique’s progress to reaching HIV epidemic control
Source: PLoS One. 2026 Jul 29;21(7):e0354368. doi: 10.1371/journal.pone.0354368 (PMC13419232; doi:10.1371/journal.pone.0354368)
Supplement: Attachment — Submitted filename: PONE Round 2 Response to Reviewers 26062026.pdf [file pone.0354368.s005.pdf]

PONE-D-26-12452R1

How antiretroviral data cleaning reshaped Mozambique's progress to reaching HIV epidemic control

June 26, 2026

Dear PLOS One Editors,

Thank you for your review, please find your questions/comments and the following responses highlighted in **bold/green.**

With Regards,

Orrin Tiberi

Additional Editor Comments:

Dear Authors,

Thank you for putting your important work together for other jurisdictions to learn from. You have made improvements to the manuscript in response to reviewer comments. To be able to move this manuscript to the next stage, I invite you to address the outstanding comment by Reviewer 1.
Indeed some sort of treatment and corresponding discussion of the Provinces will be necessary for readers to understand why there is a difference between them - are there predominantly rural Provinces vs predominantly urban? Other factors such as staffing levels, frequency of supervision which may be related to distance from capital cities of provinces, availability of electronic medical records and other data management infrastructure, etc. This further analysis as much as available data allows will improve your manuscript. **– thank you for the comment. This has been included in the discussion on lines 195-199. As this question was not part of the data cleaning activity, we don't have exact reasons why these differences may have occurred, however have included a few that we have noted anecdotally as impactful during the activity.**

Reviewers' comments:

1. The authors stated in their response to my previous comments that they could not build/recover an important variable (urban/rural). This is a serious limitation and should be recognized as such. **– Thank you for your comment. With the adjustments made for the second question we believe this is no longer a structural limitation of the manuscript/analysis.**

2. It is stated in the limitation that "In contrast, rural and smaller health facilities without donor support, which make up most of the health facilities with ART services, did not have regular supervision visits for validation despite recording the largest differences in the analysis". Two questions:

- How do you know they are rural, and yet you cannot perform an analysis stratifying urban and rural? For whatever health facilities you managed to have classified as

urban/rural based on the SARA information, it is possible to add to tables 1 and 2 such an analysis (overall urban, rural, and missing). **– Thank you for your comment. We can estimate the rural and urban division through a few methods. In the Mozambique Demographic and Health Survey 2022-23, the rural/urban divide of the population is approx. 65% rural/35% urban. Furthermore, the Service Availability and Readiness Assessment (SARA) included 1,360 health facilities (HFs), of which 229 are classified as urban and 1,131 as rural. However, because SARA data represent approximately 77% of the total number of HFs reported at the end of 2024, we believe that presenting these data directly in the manuscript may introduce additional questions regarding representativeness without providing actionable information for interpretation of the study findings. As an alternative, we have incorporated a proxy measure that more directly reflects the characteristics of the facilities included in our analysis. In 2018, PEPFAR categorized health facilities in Mozambique as AJUDA and non-AJUDA sites. AJUDA-supported facilities were defined as those with more than 500 PLHIV on ART and were predominantly located in urban or peri-urban settings. This distinction is represented in Table 1 through the indicator "Presence of EMR at the HF," with 658 facilities having an electronic medical record (EMR) system and 1,094 without EMR. We have also added the number of health facilities by volume category to Table 1. The distribution of facility size, low volume (n=944), medium volume (n=268), and high volume (n=545), further demonstrates the alignment between facility volume, EMR availability, and the likely urban/peri-urban concentration of higher-volume facilities. Furthermore, because EMR availability is concentrated among higher volume facilities, which are primarily urban and peri-urban sites, analyses based on EMR status would capture many of the same underlying differences in service delivery capacity for the rural/urban analysis, including improved monitoring of ART entry, retention, and treatment outcomes. We have revised the Introduction (lines 70–71) to explicitly acknowledge the urban and higher-volume focus of EMR-supported facilities and hope that these adjustments address the concern raised.**

- Does this mean there are health facilities without supervised validation? **– thank you for the comment, and yes not all facilities received physical supervision due to the large number of facilities in the country (~1,750) Despite this, the national level provided virtual supervision on all facilities by weekly monitoring of extreme drops or increases in PLHIV on ART, which were then prioritized for potential supervision visits. Additionally provincial and district government personnel were responsible for conducting supervisions, applying the same methodology as the central level, and the list of HF that received central level validation were randomly selected based off size categories (3 high volume, 2 med, 1 low)**

3. Please revise the title in the results section on line 148. Change "Results between…" to "Comparison between…" or some better designation. **– thank you, this has been updated in accordance with your suggestion**

4. Is it possible to provide a figure illustrating both the steps for data processing and the validation? **– Thank you for the suggestion. We have included a data flow on line 99**
