# Supplementary material for: How antiretroviral data cleaning reshaped Mozambique’s progress to reaching HIV epidemic control
Source: PLoS One. 2026 Jul 29;21(7):e0354368. doi: 10.1371/journal.pone.0354368 (PMC13419232; doi:10.1371/journal.pone.0354368)
Supplement: Peer review history

# Peer review: Decision Letter 0

**DOI:** 10.1371/journal.pone.0354368.r001

**Authors:** Edward Oladele

23 Apr 2026

PONE-D-26-12452

How antiretroviral data cleaning reshaped Mozambique’s progress to reaching HIV epidemic control

PLOS One

Dear Dr. Tiberi,

Thank you for submitting your manuscript to PLOS ONE. After careful consideration, we feel that it has merit but does not fully meet PLOS ONE’s publication criteria as it currently stands. Therefore, we invite you to submit a revised version of the manuscript that addresses the points raised during the review process.

Please submit your revised manuscript by Jun 07 2026 11:59PM. If you will need more time than this to complete your revisions, please reply to this message or contact the journal office at plosone@plos.org. When you're ready to submit your revision, log on to https://www.editorialmanager.com/pone/ and select the 'Submissions Needing Revision' folder to locate your manuscript file.

Please include the following items when submitting your revised manuscript:

- A letter that responds to each point raised by the academic editor and reviewer(s). You should upload this letter as a separate file labeled 'Response to Reviewers'.
- A marked-up copy of your manuscript that highlights changes made to the original version. You should upload this as a separate file labeled 'Revised Manuscript with Track Changes'.
- An unmarked version of your revised paper without tracked changes. You should upload this as a separate file labeled 'Manuscript'.

If you would like to make changes to your financial disclosure, please include your updated statement in your cover letter. Guidelines for resubmitting your figure files are available below the reviewer comments at the end of this letter.

If applicable, we recommend that you deposit your laboratory protocols in protocols.io to enhance the reproducibility of your results. Protocols.io assigns your protocol its own identifier (DOI) so that it can be cited independently in the future. For instructions see: https://journals.plos.org/plosone/s/submission-guidelines#loc-laboratory-protocols. Additionally, PLOS ONE offers an option for publishing peer-reviewed Lab Protocol articles, which describe protocols hosted on protocols.io. Read more information on sharing protocols at https://plos.org/protocols?utm_medium=editorial-email&utm_source=authorletters&utm_campaign=protocols.

As the corresponding author, your ORCID iD is verified in the submission system and will appear in the published article. PLOS supports the use of ORCID, and we encourage all coauthors to register for an ORCID iD and use it as well. Please encourage your coauthors to verify their ORCID iD within the submission system before final acceptance, as unverified ORCID iDs will not appear in the published article. *Only*  the individual author can complete the verification step; PLOS staff *cannot*  verify ORCID iDs on behalf of authors.

We look forward to receiving your revised manuscript.

Kind regards,

Edward Adekola Oladele, MD, MPH, PhD

Academic Editor

PLOS One

Journal Requirements:

When submitting your revision, we need you to address these additional requirements.

1.Please ensure that your manuscript meets PLOS ONE's style requirements, including those for file naming. The PLOS ONE style templates can be found at

https://journals.plos.org/plosone/s/file?id=wjVg/PLOSOne_formatting_sample_main_body.pdf and

https://journals.plos.org/plosone/s/file?id=ba62/PLOSOne_formatting_sample_title_authors_affiliations.pdf

2. Thank you for stating the following financial disclosure:

“This study was supported by the US President’s Emergency Plan for AIDS Relief (PEPFAR) through the Centers for Disease Control and Prevention (CDC) under the terms of GGH002520.”

Please state what role the funders took in the study.  If the funders had no role, please state: "The funders had no role in study design, data collection and analysis, decision to publish, or preparation of the manuscript."

If this statement is not correct you must amend it as needed.

Please include this amended Role of Funder statement in your cover letter; we will change the online submission form on your behalf.

3. Please include captions for your Supporting Information files at the end of your manuscript, and update any in-text citations to match accordingly. Please see our Supporting Information guidelines for more information: http://journals.plos.org/plosone/s/supporting-information.

4. Thank you for providing your underlying data as Supporting Information.

We note that the data set contains text or data that is not in English. Please note that PLOS is an English-language publisher, so we require data sets to be provided in English as well. Please upload an English-language version of your data set.

This will also allow us to determine if your data follows PLOS standards per our Data Availability policy here: https://journals.plos.org/plosone/s/data-availability

5. If the reviewer comments include a recommendation to cite specific previously published works, please review and evaluate these publications to determine whether they are relevant and should be cited. There is no requirement to cite these works unless the editor has indicated otherwise.

6. Please review your reference list to ensure that it is complete and correct. If you have cited papers that have been retracted, please include the rationale for doing so in the manuscript text, or remove these references and replace them with relevant current references. Any changes to the reference list should be mentioned in the rebuttal letter that accompanies your revised manuscript. If you need to cite a retracted article, indicate the article’s retracted status in the References list and also include a citation and full reference for the retraction notice.

[Note: HTML markup is below. Please do not edit.]

Reviewers' comments:

Reviewer's Responses to Questions

**Comments to the Author**

1. Is the manuscript technically sound, and do the data support the conclusions?

The manuscript must describe a technically sound piece of scientific research with data that supports the conclusions. Experiments must have been conducted rigorously, with appropriate controls, replication, and sample sizes. The conclusions must be drawn appropriately based on the data presented.

Reviewer #1: Yes

Reviewer #2: Yes

**********

2. Has the statistical analysis been performed appropriately and rigorously?

Reviewer #1: Yes

Reviewer #2: Yes

**********

3. Have the authors made all data underlying the findings in their manuscript fully available?

The PLOS Data policy requires authors to make all data underlying the findings described in their manuscript fully available without restriction, with rare exception (please refer to the Data Availability Statement in the manuscript PDF file). The data should be provided as part of the manuscript or its supporting information, or deposited to a public repository. For example, in addition to summary statistics, the data points behind means, medians and variance measures should be available. If there are restrictions on publicly sharing data—e.g. participant privacy or use of data from a third party—those must be specified.

Reviewer #1: Yes

Reviewer #2: Yes

**********

4. Is the manuscript presented in an intelligible fashion and written in standard English?

PLOS ONE does not copyedit accepted manuscripts, so the language in submitted articles must be clear, correct, and unambiguous. Any typographical or grammatical errors should be corrected at revision, so please note any specific errors here.

Reviewer #1: Yes

Reviewer #2: Yes

**********

5. Review Comments to the Author

Please use the space provided to explain your answers to the questions above. You may also include additional comments for the author, including concerns about dual publication, research ethics, or publication ethics. (Please upload your review as an attachment if it exceeds 20,000 characters)

Reviewer #1: The authors have done well to document a commonly observed and topical phenomenon in ART programs in sub-Saharan Africa. The methods have been well elaborated, and the findings well discussed. The authors' reflections provide further insight into the implications of the findings for practice and policy. However, I have the following recommendations to further increase the understanding and improve reproducibility of the study.

INTRODUCTION

Line 50: Provide one sentence on what the 95-95-95 targets are to improve ease of understanding for persons not previously familiar with the HIV program.

METHODS

Line 78: Replace the word "among" with "across"

Lines 81-82: Please clarify whether the clients' current age computed, or the age as documented in the clinical records as of the time of enrollment into ART were captured?

DATA ANALYSIS

Line 96: Was the <=59 days threshold based on the last expected pickup visit or the last documented pickup visit? Please be explicit about the criteria.

HIV ESTIMATES

Line 111: Replace the word "different" with "difference".

RESULTS

Line 131: Tautology, please revise sentence.

Table 1 - In your reflections, please suggest that future studies may explore what informed the differences in the error rates across provinces. One would have expected provinces with higher client loads to have higher error rates. But this is not established with the results. Perhaps a qualitative study or data management process evaluation could unravel further insight.

Table 1 - You may also explore using some inferential statistics like T-test or Mann Whitney U test depending on the data distribution to establish the nuanced differences in error rates by age, sex, provinces, facility client load, and presence of EMR.

Lines 140-142: In your reflections, please provide practical recommendations for unifying the reporting system so that there is one single source of truth for PEPFAR and MOH data going forward?

Table 2 - In your discussions or reflections please expatiate on why there are still differences in PLHIV on ART between PEPFAR and MOH records post-data cleaning. One would have expected the same cleaned data to feed both information systems. This was not very clear in your methodology section also, so it raises questions in when this is presented in Table 2 results.

Reviewer #2: This is an important report for Mozambique as well as for many other global settings with high HIV burden. Here, the authors quantify the discrepancy in the number of PLHIV between PEPFAR counts and the Ministry of Health (MOH) in Mozambique before and after a data cleaning exercise. The “before” comparison confirms the important discrepancy. The “after” comparison shows a reduction in discrepancy (an improvement but with persistent discrepancies) as well as important implications in the model-based forecast.

Few issues:

1. In the abstract and the methods, I would suggest reformulating the first sentence in the methods to avoid the word “triangulated”. At this stage, not many readers understand that triangulation involves using multiple data sources to verify accuracy and consistency. In fact, in the main text, there is an overuse of the word “triangulation” without ever presenting a definition or some sort of specification in this particular study/analysis.

Also, I would suggest removing the mention of ART coverage in the discussion (in the abstract). Put the number of people living with HIV under ART.

2. The introduction is well written and makes a good case of why it matters to do this study.

Lines 55/58 - Please do revise in light of the events last year.

Line 61 - Please confirm citation 7 for the number of health facilities of high-volume or how the MoH aggregates the data. This is because citation 7 seems to be a clinical guide.

3. Methods:

- Please set a sort of definition of what triangulation means in this work

- It would be helpful to have a figure describing how the triangulation (checking, validation, confirmation, etc.) algorithm is done.

- Data analysis: Two things about the age. i) What age is used here? Is this age at ART registration or age at the time of the survey, or another sort of age? Please specify. ii) Age group divisions. It would be good if the age groups are under-5, 5 to 9, 10 to 14, 15 to 19, 20 to 35, and 35 or more. If not possible, please give a justification for the groups chosen.

- HIV Estimates: Perhaps the title of this subsection should be “HIV forecast estimates”. Or choose a title that indicates that some mathematical modeling is used.

- HIV Estimates: Lines 110/115 - please rephrase to make it clearer. What are the weights used for? And how were they chosen?

- About the absolute differences: The absolute differences are a good indicator. But they focus only on the magnitude of the difference and ignore its direction. It matters here to know both.

- About the relative differences: Please indicate how the relative differences are computed. In this, it should be clear which reference (MoH or PEPFAR counts) is used and whether it can be negative or positive.

4. For the results:

- It would be good to have a frequency table with the reasons why records were removed or added. What did the “triangulation” lead to 222,737 fewer observations?

- Table 1 - Indicate in a footnote how the percent change is calculated.

- Table 1: Is it possible to repeat this analysis stratified by urban/rural health facilities?

- Table 2: We need rural/urban analysis as well. Please indicate in the footnotes how the relative differences are computed.

- Table 3: Please indicate that these are Spectrum estimates

**********

6. PLOS authors have the option to publish the peer review history of their article (what does this mean?). If published, this will include your full peer review and any attached files.

If you choose “no”, your identity will remain anonymous but your review may still be made public.

**Do you want your identity to be public for this peer review?**  For information about this choice, including consent withdrawal, please see our Privacy Policy.

Reviewer #1: **Yes:**  Oluwasanmi Akanji Adedokun

Reviewer #2: No

**********

[NOTE: If reviewer comments were submitted as an attachment file, they will be attached to this email and accessible via the submission site. Please log into your account, locate the manuscript record, and check for the action link "View Attachments". If this link does not appear, there are no attachment files.]

To ensure your figures meet our technical requirements, please review our figure guidelines: https://journals.plos.org/plosone/s/figures

You may also use PLOS’s free figure tool, NAAS, to help you prepare publication quality figures: https://journals.plos.org/plosone/s/figures#loc-tools-for-figure-preparation.

NAAS will assess whether your figures meet our technical requirements by comparing each figure against our figure specifications.

Attachment Submitted filename: PONE-D-26-12452.pdf

# Author comment: Author response to Decision Letter 1

**DOI:** 10.1371/journal.pone.0354368.r002

14 May 2026

Thank you for the review and comments. As stated above, the majority of the comments were clear and has allowed us to achieve a stronger submission. I have uploaded a line by line response as part of the submission for your review. Thank you again,

Attachment Submitted filename: PONE Round 1 Reviewer Resposes 12052026.pdf

# Peer review: Decision Letter 1

**DOI:** 10.1371/journal.pone.0354368.r003

**Authors:** Edward Oladele

24 Jun 2026

PONE-D-26-12452R1

How antiretroviral data cleaning reshaped Mozambique’s progress to reaching HIV epidemic control

PLOS One

Dear Dr. Tiberi,

Thank you for submitting your manuscript to PLOS ONE. After careful consideration, we feel that it has merit but does not fully meet PLOS ONE’s publication criteria as it currently stands. Therefore, we invite you to submit a revised version of the manuscript that addresses the points raised during the review process.

Please submit your revised manuscript by Aug 08 2026 11:59PM. If you will need more time than this to complete your revisions, please reply to this message or contact the journal office at plosone@plos.org. When you're ready to submit your revision, log on to https://www.editorialmanager.com/pone/ and select the 'Submissions Needing Revision' folder to locate your manuscript file.

Please include the following items when submitting your revised manuscript:

- A letter that responds to each point raised by the academic editor and reviewer(s). You should upload this letter as a separate file labeled 'Response to Reviewers'.
- A marked-up copy of your manuscript that highlights changes made to the original version. You should upload this as a separate file labeled 'Revised Manuscript with Track Changes'.
- An unmarked version of your revised paper without tracked changes. You should upload this as a separate file labeled 'Manuscript'.

If you would like to make changes to your financial disclosure, please include your updated statement in your cover letter. Guidelines for resubmitting your figure files are available below the reviewer comments at the end of this letter.

If applicable, we recommend that you deposit your laboratory protocols in protocols.io to enhance the reproducibility of your results. Protocols.io assigns your protocol its own identifier (DOI) so that it can be cited independently in the future. For instructions see: https://journals.plos.org/plosone/s/submission-guidelines#loc-laboratory-protocols. Additionally, PLOS ONE offers an option for publishing peer-reviewed Lab Protocol articles, which describe protocols hosted on protocols.io. Read more information on sharing protocols at https://plos.org/protocols?utm_medium=editorial-email&utm_source=authorletters&utm_campaign=protocols.

As the corresponding author, your ORCID iD is verified in the submission system and will appear in the published article. PLOS supports the use of ORCID, and we encourage all coauthors to register for an ORCID iD and use it as well. Please encourage your coauthors to verify their ORCID iD within the submission system before final acceptance, as unverified ORCID iDs will not appear in the published article. *Only*  the individual author can complete the verification step; PLOS staff *cannot*  verify ORCID iDs on behalf of authors.

We look forward to receiving your revised manuscript.

Kind regards,

Edward Adekola Oladele, MD, MPH, PhD

Academic Editor

PLOS One

Journal Requirements:

If the reviewer comments include a recommendation to cite specific previously published works, please review and evaluate these publications to determine whether they are relevant and should be cited. There is no requirement to cite these works unless the editor has indicated otherwise.

Please review your reference list to ensure that it is complete and correct. If you have cited papers that have been retracted, please include the rationale for doing so in the manuscript text, or remove these references and replace them with relevant current references. Any changes to the reference list should be mentioned in the rebuttal letter that accompanies your revised manuscript. If you need to cite a retracted article, indicate the article’s retracted status in the References list and also include a citation and full reference for the retraction notice.

**Additional Editor Comments:**

Dear Authors,

Thank you for putting your important work together for other jurisdictions to learn from. You have made improvements to the manuscript in response to reviewer comments. To be able to move this manuscript to the next stage, I invite you to address the outstanding comment by Reviewer 1.

Indeed some sort of treatment and corresponding discussion of the Provinces will be necessary for readers to understand why there is a difference between them - are there predominantly rural Provinces vs predominantly urban? Other factors such as staffing levels, frequency of supervision which may be related to distance from capital cities of provinces, availability of electronic medical records and other data management infrastructure, etc. This further analysis as much as available data allows will improve your manuscript.

[Note: HTML markup is below. Please do not edit.]

Reviewers' comments:

Reviewer's Responses to Questions

**Comments to the Author**

1. If the authors have adequately addressed your comments raised in a previous round of review and you feel that this manuscript is now acceptable for publication, you may indicate that here to bypass the “Comments to the Author” section, enter your conflict of interest statement in the “Confidential to Editor” section, and submit your "Accept" recommendation.

Reviewer #1: All comments have been addressed

Reviewer #2: (No Response)

**********

2. Is the manuscript technically sound, and do the data support the conclusions?

The manuscript must describe a technically sound piece of scientific research with data that supports the conclusions. Experiments must have been conducted rigorously, with appropriate controls, replication, and sample sizes. The conclusions must be drawn appropriately based on the data presented.

Reviewer #1: Yes

Reviewer #2: Yes

**********

3. Has the statistical analysis been performed appropriately and rigorously?

Reviewer #1: Yes

Reviewer #2: Yes

**********

4. Have the authors made all data underlying the findings in their manuscript fully available?

The PLOS Data policy requires authors to make all data underlying the findings described in their manuscript fully available without restriction, with rare exception (please refer to the Data Availability Statement in the manuscript PDF file). The data should be provided as part of the manuscript or its supporting information, or deposited to a public repository. For example, in addition to summary statistics, the data points behind means, medians and variance measures should be available. If there are restrictions on publicly sharing data—e.g. participant privacy or use of data from a third party—those must be specified.

Reviewer #1: Yes

Reviewer #2: Yes

**********

5. Is the manuscript presented in an intelligible fashion and written in standard English?

PLOS ONE does not copyedit accepted manuscripts, so the language in submitted articles must be clear, correct, and unambiguous. Any typographical or grammatical errors should be corrected at revision, so please note any specific errors here.

Reviewer #1: Yes

Reviewer #2: Yes

**********

6. Review Comments to the Author

Please use the space provided to explain your answers to the questions above. You may also include additional comments for the author, including concerns about dual publication, research ethics, or publication ethics. (Please upload your review as an attachment if it exceeds 20,000 characters)

Reviewer #1: The authors have taken cognizance of my previous comments, my comments have been properly addressed, and I am satisfied with the authors' responses.

Reviewer #2: This is an important manuscript reporting a validation process and results on the status of the HIV epidemic control in Mozambique. The authors responded to all the issues I raised in the previous revision. However, there remain some issues to resolve:

1. The authors stated in their response to my previous comments that they could not build/recover an important variable (urban/rural). This is a serious limitation and should be recognized as such.

2. It is stated in the limitation that “In contrast, rural and smaller health facilities without donor support, which make up most of the health facilities with ART services, did not have regular supervision visits for validation despite recording the largest differences in the analysis”. Two questions:

- How do you know they are rural, and yet you cannot perform an analysis stratifying urban and rural? For whatever health facilities you managed to have classified as urban/rural based on the SARA information, it is possible to add to tables 1 and 2 such an analysis (overall urban, rural, and missing).

- Does this mean there are health facilities without supervised validation?

3. Please revise the title in the results section on line 148. Change “Results between…” to “Comparison between…” or some better designation.

4. Is it possible to provide a figure illustrating both the steps for data processing and the validation?

**********

7. PLOS authors have the option to publish the peer review history of their article (what does this mean?). If published, this will include your full peer review and any attached files.

If you choose “no”, your identity will remain anonymous but your review may still be made public.

**Do you want your identity to be public for this peer review?**  For information about this choice, including consent withdrawal, please see our Privacy Policy.

Reviewer #1: **Yes:**  Oluwasanmi Akanji Adedokun

Reviewer #2: No

**********

[NOTE: If reviewer comments were submitted as an attachment file, they will be attached to this email and accessible via the submission site. Please log into your account, locate the manuscript record, and check for the action link "View Attachments". If this link does not appear, there are no attachment files.]

To ensure your figures meet our technical requirements, please review our figure guidelines: https://journals.plos.org/plosone/s/figures

You may also use PLOS’s free figure tool, NAAS, to help you prepare publication quality figures: https://journals.plos.org/plosone/s/figures#loc-tools-for-figure-preparation.

NAAS will assess whether your figures meet our technical requirements by comparing each figure against our figure specifications.

# Author comment: Author response to Decision Letter 2

**DOI:** 10.1371/journal.pone.0354368.r004

30 Jun 2026

Below is the same information included in the Respose to Reviewer document. Thank you.

Indeed some sort of treatment and corresponding discussion of the Provinces will be necessary for readers to understand why there is a difference between them - are there predominantly rural Provinces vs predominantly urban? Other factors such as staffing levels, frequency of supervision which may be related to distance from capital cities of provinces, availability of electronic medical records and other data management infrastructure, etc. This further analysis as much as available data allows will improve your manuscript. – thank you for the comment. This has been included in the discussion on lines 195-199. As this question was not part of the data cleaning activity, we don’t have exact reasons why these differences may have occurred, however have included a few that we have noted anecdotally as impactful during the activity.

1. The authors stated in their response to my previous comments that they could not build/recover an important variable (urban/rural). This is a serious limitation and should be recognized as such. – Thank you for your comment. With the adjustments made for the second question we believe this is no longer a structural limitation of the manuscript/analysis.

2. It is stated in the limitation that “In contrast, rural and smaller health facilities without donor support, which make up most of the health facilities with ART services, did not have regular supervision visits for validation despite recording the largest differences in the analysis”. Two questions:

• How do you know they are rural, and yet you cannot perform an analysis stratifying urban and rural? For whatever health facilities you managed to have classified as urban/rural based on the SARA information, it is possible to add to tables 1 and 2 such an analysis (overall urban, rural, and missing). – Thank you for your comment. We can estimate the rural and urban division through a few methods. In the Mozambique Demographic and Health Survey 2022-23, the rural/urban divide of the population is approx. 65% rural/35% urban. Furthermore, the Service Availability and Readiness Assessment (SARA) included 1,360 health facilities (HFs), of which 229 are classified as urban and 1,131 as rural. However, because SARA data represent approximately 77% of the total number of HFs reported at the end of 2024, we believe that presenting these data directly in the manuscript may introduce additional questions regarding representativeness without providing actionable information for interpretation of the study findings. As an alternative, we have incorporated a proxy measure that more directly reflects the characteristics of the facilities included in our analysis. In 2018, PEPFAR categorized health facilities in Mozambique as AJUDA and non-AJUDA sites. AJUDA-supported facilities were defined as those with more than 500 PLHIV on ART and were predominantly located in urban or peri-urban settings. This distinction is represented in Table 1 through the indicator “Presence of EMR at the HF,” with 658 facilities having an electronic medical record (EMR) system and 1,094 without EMR. We have also added the number of health facilities by volume category to Table 1. The distribution of facility size, low volume (n=944), medium volume (n=268), and high volume (n=545), further demonstrates the alignment between facility volume, EMR availability, and the likely urban/peri-urban concentration of higher-volume facilities. Furthermore, because EMR availability is concentrated among higher volume facilities, which are primarily urban and peri-urban sites, analyses based on EMR status would capture many of the same underlying differences in service delivery capacity for the rural/urban analysis, including improved monitoring of ART entry, retention, and treatment outcomes. We have revised the Introduction (lines 70–71) to explicitly acknowledge the urban and higher-volume focus of EMR-supported facilities and hope that these adjustments address the concern raised.

• Does this mean there are health facilities without supervised validation? – thank you for the comment, and yes not all facilities received physical supervision due to the large number of facilities in the country (~1,750) Despite this, the national level provided virtual supervision on all facilities by weekly monitoring of extreme drops or increases in PLHIV on ART, which were then prioritized for potential supervision visits. Additionally provincial and district government personnel were responsible for conducting supervisions, applying the same methodology as the central level, and the list of HF that received central level validation were randomly selected based off size categories (3 high volume, 2 med, 1 low)

3. Please revise the title in the results section on line 148. Change “Results between…” to “Comparison between…” or some better designation. – thank you, this has been updated in accordance with your suggestion

4. Is it possible to provide a figure illustrating both the steps for data processing and the validation? – Thank you for the suggestion. We have included a data flow on line 99

Attachment Submitted filename: PONE Round 2 Response to Reviewers 26062026.pdf

# Editor report: Decision Letter 2

**DOI:** 10.1371/journal.pone.0354368.r005

**Authors:** Edward Oladele

8 Jul 2026

How antiretroviral data cleaning reshaped Mozambique’s progress to reaching HIV epidemic control

PONE-D-26-12452R2

Dear Dr. Tiberi,

We’re pleased to inform you that your manuscript has been judged scientifically suitable for publication and will be formally accepted for publication once it meets all outstanding technical requirements.

Within one week, you’ll receive an e-mail detailing the required amendments. When these have been addressed, you’ll receive a formal acceptance letter and your manuscript will be scheduled for publication.

An invoice will be generated when your article is formally accepted. Please note, if your institution has a publishing partnership with PLOS and your article meets the relevant criteria, all or part of your publication costs will be covered. Please make sure your user information is up-to-date by logging into Editorial Manager at Editorial Manager® and clicking the ‘Update My Information' link at the top of the page. For questions related to billing, please contact billing support.

If your institution or institutions have a press office, please notify them about your upcoming paper to help maximize its impact. If they’ll be preparing press materials, please inform our press team as soon as possible -- no later than 48 hours after receiving the formal acceptance. Your manuscript will remain under strict press embargo until 2 pm Eastern Time on the date of publication. For more information, please contact onepress@plos.org.

Kind regards,

Edward Adekola Oladele, MD, MPH, PhD

Academic Editor

PLOS One

Additional Editor Comments (optional):

Reviewers' comments:

# Editor report: Acceptance letter

**DOI:** 10.1371/journal.pone.0354368.r006

**Authors:** Edward Oladele

PONE-D-26-12452R2

PLOS One

Dear Dr. Tiberi,

I'm pleased to inform you that your manuscript has been deemed suitable for publication in PLOS One. Congratulations! Your manuscript is now being handed over to our production team.

At this stage, our production department will prepare your paper for publication. This includes ensuring the following:

* All references, tables, and figures are properly cited

* All relevant supporting information is included in the manuscript submission,

* There are no issues that prevent the paper from being properly typeset

You will receive further instructions from the production team, including instructions on how to review your proof when it is ready. Please keep in mind that we are working through a large volume of accepted articles, so please give us a few days to review your paper and let you know the next and final steps.

Lastly, if your institution or institutions have a press office, please let them know about your upcoming paper now to help maximize its impact. If they'll be preparing press materials, please inform our press team within the next 48 hours. Your manuscript will remain under strict press embargo until 2 pm Eastern Time on the date of publication. For more information, please contact onepress@plos.org.

You will receive an invoice from PLOS for your publication fee after your manuscript has reached the completed accept phase. If you receive an email requesting payment before acceptance or for any other service, this may be a phishing scheme. Learn how to identify phishing emails and protect your accounts at https://explore.plos.org/phishing.

If we can help with anything else, please email us at customercare@plos.org.

Thank you for submitting your work to PLOS ONE and supporting open access.

Kind regards,

PLOS ONE Editorial Office Staff

on behalf of

Dr. Edward Adekola Oladele

Academic Editor

PLOS One
